# Comparative genomics identifies small interfering RNA with activity against all five human betacoronaviruses

**DOI:** 10.1186/s13073-026-01659-1

**Published:** 2026-04-22

**Authors:** Julian Vogler, Shubhankar Ambike, Stoyan Velkov, Cho-Chin Cheng, Pauline Neubecker, Natalie Fischhaber, Pratik Mallick, Ivana Martan, Lucie Sauerhering, Albrecht von Brunn, Ulrike Protzer, Thomas Michler

**Affiliations:** 1 Institute of Laboratory Medicine, LMU University Hospital, LMU Munich, Munich, Germany; 2 Institute of Virology, School of Medicine and Health, Technical University of Munich, Helmholtz Munich, Munich, Germany; 3 Institute of Virology, Philipps University Marburg, Marburg, Germany; 4 Max von Pettenkofer Institute and Gene Center, LMU Munich, Virology, Munich, Germany; 5 German Center for Infection Research (DZIF), Munich partner site, Munich, Germany; 6 Current affiliation: Program in Emerging Infectious Diseases, Duke-NUS Medical School, Singapore, Singapore

**Keywords:** Comparative genomics, *Orthocoronavirinae*, Coronavirus, RNA interference, siRNA, Broad-spectrum antiviral therapy, Pandemic preparedness

## Abstract

**Background:**

The *Orthocoronavirinae* virus subfamily (CoV) poses a continuous global health threat. Seven CoV species are known to infect humans and additional animal CoVs with potential for human spillover have been identified. Currently, antiviral drugs are available only against SARS-CoV-2, underscoring the need for broad-spectrum antivirals. Targeting the viral RNA genome with small interfering RNAs (siRNAs) induces its degradation by endogenous nucleases and represents an effective strategy to inhibit CoV replication. Previous studies have demonstrated the efficacy and feasibility of this approach against individual CoV species. However, its potential to serve as a broad-spectrum antiviral strategy effective across multiple members of the CoV subfamily has not been systematically explored.

**Methods:**

The conservation of all potential siRNA target sites within the CoV genome (*n* ≈ 30,000) was analyzed using a custom database comprising 250,000 full-length genome sequences from 44 CoV species isolated from diverse mammalian and avian hosts. Antiviral activity of selected siRNA candidates was screened using a replication-competent recombinant SARS-CoV-2 expressing green fluorescent protein. The effect of target site mismatches was assessed using luciferase reporters, and cross-species antiviral activity was evaluated against six representative wild-type CoVs. Cellular toxicity was investigated by monitoring cell death, confluence, metabolic activity, and in silico prediction of siRNA off-target activity.

**Results:**

Conservation of potential siRNA target sites correlated with the evolutionary distance between CoV species. Several genomic regions were conserved across different CoV subgenera and genera, but no site was universally preserved across all 44 analyzed species. The antiviral activity and tolerability of 347 siRNAs targeting the most promising sites were evaluated in multiple screening rounds. Following optimization of siRNA design and chemistry, we identified a lead candidate, si117m, which displayed high tolerability and potent silencing activity at picomolar concentrations against target sites of SARS-CoV-1, SARS-CoV-2, MERS-CoV, HCoV-OC43 and HCoV-HKU1.

**Conclusions:**

Our study demonstrates the potential of comparative genomics for developing broad-spectrum antiviral siRNAs. We identified a lead siRNA, si117m, which combines a favorable safety profile with cross-species activity against five human CoVs, supporting its potential for clinical translation and future pandemic preparedness.

**Supplementary Information:**

The online version contains supplementary material available at 10.1186/s13073-026-01659-1.

## Background

The subfamily of *Orthocoronavirinae* (`Coronavirus´ = CoV) has caused immense human suffering. It is classified into four genera (*Alpha-*,* Beta-*,* Gamma-*,* and Deltacoronavirus*), which are further divided into 26 subgenera, comprising approximately 50 species in total [1]. CoVs infect a broad range of hosts, encompassing numerous mammalian and avian species [2]. The pathology caused by each species varies in terms of the affected organs and the disease manifestation. While CoVs primarily cause respiratory disease in humans and birds, they predominantly affect the gastrointestinal tract in non-primate mammals, including dogs, cattle, pigs or cats [2].

To date, seven CoVs have been identified to infect humans (human CoVs = HCoVs). The so-called `seasonal´ HCoVs comprise HCoV-OC43, -NL63, -229E, and -HKU1 and typically cause mild cold-like symptoms [3]. Although seasonal CoVs are thought to have originated in animal reservoirs, transmission into the human population likely occurred at least a century ago [4]. In contrast, three HCoVs, all belonging to the *Betacoronavirus* genus, have more recently crossed into the human population from animal reservoirs. At least in part due to low or absent immunity in the human population, these viruses tend to cause more severe illness and spread rapidly among humans, leading to epidemics or even pandemics (pandemic CoVs) [5]. This group includes the severe acute respiratory syndrome CoV (SARS-CoV), which caused a self-limiting epidemic in 2003 with an estimated case fatality rate of 10% [6]. Nine years later, the Middle East Respiratory Syndrome CoV (MERS-CoV) emerged. Although human-to-human transmission of MERS-CoV is rare, infected individuals have a high mortality rate (approximately 34%), with dromedary camels serving as the primary source of transmission [7]. More recently, the spillover of SARS-CoV-2, the causative pathogen of COVID-19, resulted in a pandemic that led to more than 7 million deaths worldwide as of August 2025 [8] and a 10% decline of global GDP [9]. The crossing of three highly pathogenic CoVs from animal reservoirs into the human population within the last 22 years underscores the ongoing risk of future zoonotic events. Alarmingly, it has already been demonstrated that additional CoVs, which are currently confined to animal reservoirs, have the potential to replicate in human cells [10, 11], highlighting the risk of novel pandemic CoVs to arise in the future.

Despite the serious health burden and risks posed by CoVs, antiviral drugs remain limited. Indeed, antiviral therapies are approved only for the treatment of COVID-19 [12] and not for diseases caused by the other six HCoVs. The narrow spectrum of activity raises concerns regarding global preparedness for future CoV pandemics and may facilitate the emergence of escape mutations that render CoVs resistant to available drugs [13, 14], as already observed for several SARS-CoV-2 drugs in recent years [15, 16]. Taken together, there is a persisting high need to develop antivirals that are broadly active against multiple CoV species, both to manage currently circulating HCoVs and to enhance preparedness for future pandemics.

One highly effective approach to inhibit replication and spread of a wide range of viruses involves the use of RNA interference (RNAi) [17–19]. RNAi is an endogenous mechanism for post-transcriptional gene regulation, by which specific RNAs are silenced or degraded in a sequence-specific manner [20]. This mechanism can be therapeutically exploited to silence harmful RNA, such as the genomes of RNA viruses, by introducing synthetic small interfering RNA (siRNA) into cells. These siRNAs are double-stranded and typically 19 to 25 nucleotides (nt) in length [21]. The so-called antisense strand hybridizes with complementary viral RNA, triggering its cleavage by the RNA-induced silencing complex (RISC) [22]. RNAi-based therapeutics have gained substantial traction in recent years, with several siRNAs already approved for clinical use [23]. These successes have validated the clinical utility of siRNAs and paved the way for their application in antiviral therapy. A notable example was ALN-RSV01, an siRNA that targeted the nucleocapsid gene of respiratory syncytial virus (RSV). Although a phase 2 trial did not meet al.l clinical endpoints, it provided proof-of-concept for the safety and feasibility of inhaled antiviral siRNA-based drugs, protecting 38% of participating children from RSV infection [24]. This was particularly remarkable given that ALN-RSV01 employed first-generation siRNA technology lacking chemical modifications and delivery enhancements. Since then, two technological advances have strongly improved the potential of siRNAs in general and especially in the context of respiratory diseases. First, the introduction of chemical modifications has significantly increased siRNA stability by conferring resistance to RNases and reducing immunogenicity [25, 26]. Second, multiple delivery platforms have been developed to enhance siRNA uptake in the human respiratory tract, including lipid nanoparticles (LNPs) [27], polymer-based carriers [28, 29], lipophilic conjugates such as C16-siRNAs [30], or ligand-conjugated siRNAs targeting receptors like integrin αvβ6 [31]. These innovations have enabled robust siRNA-mediated silencing in the respiratory tract, as demonstrated in advanced ex vivo models [29, 32], in vivo models [33, 34], and in humans [31].

In parallel, we and others have demonstrated that CoVs can be effectively targeted by siRNAs [29, 32, 35–39]. Using SARS-CoV-2 as a model virus, we systematically investigated which CoV replication steps and which viral RNA species are accessible to RNAi-based therapeutics. Our results demonstrated that siRNAs can target the CoV genomic RNA (gRNA) directly after cell entry, terminating replication prior to the onset of transcription and preventing virus-induced cytopathy [32].

Proof-of-concept for siRNA-mediated inhibition has been demonstrated primarily for the pandemic HCoVs SARS-CoV [35], MERS-CoV [36], and SARS-CoV-2 [29, 32, 37, 39]. However, reports of siRNAs with cross-species antiviral activity remain limited. While several studies have described targeting of different SARS-CoV-2 variants [32, 37, 40], only two studies reported that siRNA target sites identified in SARS-CoV-2 were also conserved in the closely related SARS-CoV-1 [40, 41]. To the best of our knowledge, no siRNA-based therapeutic strategy has yet demonstrated cross-species activity or even target site conservation beyond these closely related CoVs within the *Sarbecovirus* subgenus. Such cross-reactivity would be essential for treating a broader spectrum of HCoV infections and for increasing the likelihood that a single antiviral agent remains effective against future emerging pandemic strains. However, it is so far unclear whether RNAi-based therapeutics can achieve this goal or whether the high genetic variability of CoVs poses a fundamental barrier to cross-species activity beyond closely related CoV species.

In this study, we aimed to systematically evaluate the potential of siRNAs to serve as broad-spectrum antiviral agents against CoVs. To this end, we performed a comprehensive genomic analysis of the *Orthocoronavirinae* subfamily, followed by high-throughput screening for siRNA antiviral activity to identify siRNA candidates capable of suppressing multiple *Orthocoronavirinae* members with high efficacy and a favorable safety profile.

## Methods

### Database generation of full-length *Orthocoronavirinae* sequences

All available CoV sequences, including metadata, were downloaded from the NCBI Nucleotide Database [42] on February 3rd, 2021 using the search term “*Orthocoronavirinae”* without additional filters. Additionally, 200,000 sequences were retrieved from the Global Initiative on Sharing All Influenza Data (GISAID) database. Of these, 100,000 sequences were randomly sampled without selection criteria, while the remaining 100,000 included specific variants of concern and interest: Alpha (20,000), Beta (20,000), Delta (20,000), Gamma (20,000), Omicron (11,160), Lambda (848), and Mu (5,889). Sequences < 25,000 nt were excluded. Reference sequences for the different CoV species (Additional file 1: Fig. S1) were defined according to Chan et al. [43]. Sequences were allocated to specific CoV species based on Basic Local Alignment Search Tool (BLAST) analysis [44], selecting the reference sequence with the highest sequence identity. This BLAST-based assignment was used to harmonize species classification across heterogeneous NCBI annotations. All sequences used to generate the sequence database are publicly available via Zenodo at 10.5281/zenodo.18471403 [45].

### Phylogenetic trees and evolutionary distance

Full-length CoV sequences were aligned using the MAFFT v7.526 (2024/Apr/26) tool with default settings [46]. Evolutionary distances were calculated with the RaxML-NG tool [47] (v. 1.1) using the GTR + G (General time reversible and discrete Gamma) model with 10 randomized parsimony starting trees and 200 bootstrap replicates. The highest rated phylogenetic tree (bestTree) was selected and visualized using the FigTree v1.4.4. software (http://tree.bio.ed.ac.uk/software/figtree/). The evolutionary distance of each CoV species from the SARS-CoV-2 Wuhan strain was defined as the cumulative branch length along the phylogenetic tree.

### Conservation analysis of potential siRNA target sites

The 19-mer sequences were extracted from the SARS-CoV-2 Wuhan reference sequence (NC_045512.2) by sliding the 19-mer window from the 5’ to the 3’ end of the genome by a single nucleotide each time, resulting in 29,871 unique sequences. All CoV sequences in the database were screened for the presence of each of the 29,871 19-mers, each time allowing a mismatch either at position 1 or 19. The conservation within each species was defined as the proportion of sequences containing the respective 19-mer sequence.

The conservation of the target regions of the 11 lead siRNAs was analyzed at single-nucleotide resolution. For each HCoV except SARS-CoV-2, all available sequences were extracted from our database and aligned to the target sequence of the analyzed siRNA. To obtain a realistic overview over the conservation of siRNAs within globally circulating SARS-CoV-2 variants, the respective analysis was performed using a globally representative sequence alignment which was downloaded from the Nextstrain SARS-CoV-2 open dataset (*n* = 5,066 sequences) [48] on January 2nd, 2026. For our internal database of approximately 200,000 SARS-CoV-2 sequences from GISAID, sequences were screened for identity with the Wuhan reference sequence, and alignments were generated only for sequences differing from the reference. For each 21-mer target region, the conservation of individual nucleotide positions was calculated from the alignments as the fraction of sequences containing the complementary base to the siRNA antisense strand at the respective position. All sequence alignments and analysis scripts used for the conservation analysis are available via Zenodo at 10.5281/zenodo.1847140345 [45].

### siRNA design

siRNAs were designed using the SARS-CoV-2 Wuhan strain (NC_045512.2) as a template. Three additional siRNAs targeting GFP (siGFP), firefly luciferase (siLuc) or signal recognition particle 14 (siSRP14) were designed as negative controls. siLuc was used in rSARS-CoV-2^GFP^ screening experiments, siGFP in in vitro infection and luciferase assays, and siSRP14 in MERS-CoV infection experiments. An siRNA targeting the coding region of *Renilla* luciferase (siREN) was designed as positive control. Unmodified siRNA duplexes were purchased in desalted form (Microsynth AG, Balgach, Switzerland) and resuspended in RNAse-free water. Chemically modified siRNAs were purchased from Eurogentec S.A. (Seraing, Belgium) as high performance liquid chromatography (HPLC)-purified single strands and annealed following the manufacturer’s instructions.

### Cell lines and viruses

BHK21, Huh-7.5, HEK293T and A549^hACE2^ cells were maintained in Dulbecco’s modified Eagle’s medium (DMEM, high glucose, GlutaMAX™ Supplement, Pyruvat, Gibco, Thermo Fisher Scientific, Germany) supplemented with 10% fetal bovine serum (FBS) and 1% penicillin/streptomycin at 37 °C with 5% CO2. Calu-3 cells were maintained in Dulbecco’s modified Eagle’s medium/F-12 (DMEM/F-12 (1x), GlutaMAX™-I Supplement, Gibco, Thermo Fisher Scientific, Germany) supplemented with 10% FBS, 1% penicillin/streptomycin and 1% glutamine at 37 °C with 5% CO2. The rSARS-CoV-2-GFP vector was kindly provided by Prof. Dr. Volker Thiel. The genomic sequences of used CoV isolates are available under the following accession numbers: SARS-CoV-2 variant XBB.1.5 (GISAID EPI_ISL_582134), SARS-CoV-2 variant EU1 (GISAID EPI ISL: 17300038), SARS-CoV-1 (GenBank AY291315.1), MERS-CoV (GenBank JX869059), HCoV-OC43 (NCBI PX101827), HCoV-NL63 (NCBI PX112333).

### Screening antiviral activity of siRNAs using rSARS-CoV-2^GFP^

Real-time monitoring of rSARS-CoV-2^GFP^ spread was performed as previously described [32]. In brief, A549^hACE2^ cells were reverse-transfected with 10 nM siRNA using Lipofectamine RNAiMAX (Thermo Fisher Scientific, Germany) and after 6 h infected with rSARS-CoV-2^GFP^ using an MOI of 0.3. After 1 h, the inoculum was removed and replaced with fresh growth medium. Phase contrast and GFP fluorescence images of each well were captured every 4 h for 72 h using the IncuCyte^®^ Live-Cell Analysis System (Essen Bioscience, USA). GFP-positive cells were quantified using the IncuCyte S3 software (version 2019B Rev2).

### Evaluation of siRNA tolerability and prediction of off-target activity

A549^hACE2^ cells were reverse-transfected with siRNA at a 10 nM concentration and 6 h later the medium was replaced with fresh growth medium. Dead cells were quantified using the IncuCyte^®^ Cytotox Red Dye (Sartorius AG, Göttingen, Germany, Cat. No. 4632), which selectively labels cells with compromised plasma membranes. Dead cell counts were normalized to the estimated total number of cells per well to obtain the percentage of dead cells. Total cell numbers were extrapolated based on changes in confluency measured by the IncuCyte^®^ system in combination with the initial seeding density of 30,000 cells per well. The metabolic activity of cells was assessed 72 h after siRNA transfection using the CellTiter-Blue^®^ Assay (Promega, Walldorf, Germany) according to the manufacturer’s instructions and by measuring fluorescence at 590 nm wavelength using a Tecan Infinite F200 plate reader (Tecan Group Ltd., Switzerland). The potential of siRNA to cause off-target activity was predicted using the siSPOTR algorithm, which calculates the Probability of Off-Targeting Score (POTS) based on predicted seed region complementarity to the human transcriptome [49]. Lower POTS values indicate reduced off-target risk.

### Dual-Luciferase reporter plasmid design

The psiCHECK2 dual-luciferase reporter vectors used for the dual-luciferase assays were custom-synthesized by BioCat GmbH (Heidelberg, Germany). The vector inserts, cloned into the 3’UTR of *Renilla* luciferase (rLuc), comprised eight 32-mers in a concatemerized arrangement which contained the positive sense viral genomic RNA targets of the 11 siRNA lead candidates. The virus-specific inserts were designed based on the NCBI reference sequences of SARS-CoV-2 (NC_045512.2), SARS-CoV-1 (NC_004718.3), MERS-CoV (NC_038294.1), HCoV-HKU1 (NC_006577.2), HCoV-OC43 (NC_006213.1), HCoV-NL63 (NC_005831.2) and HCoV_229E (NC_002645.1).

### Dual-Luciferase reporter assay

HEK293T cells were seeded in 96 well plates at a density of 6.400 cells per well in 100 µL of growth medium without antibiotics and co-transfected after 48 h with 100 ng reporter plasmid and siRNA using Lipofectamine 2000 (Thermo Fisher Scientific, Germany; 0,5 µL per well). As a negative control, siGFP was used. Following the manufacturer’s instructions, firefly (transfection control) and *Renilla* (siRNA target) luciferase activities were assayed using the Dual-Glo™ Luciferase Assay System (Promega, Walldorf, Germany) 24 h post transfection. siRNA silencing was assessed by first normalizing the *Renilla L*uciferase signal against the firefly luciferase signal within each well and then calculated as a percentage relative to cells that were transfected with the same plasmid but no siRNA (mock). For experiments involving different reporter constructs, results were further normalized to the siREN positive control siRNA.

### Evaluation of siRNA antiviral activity against wild-type HCoVs

The antiviral activity of lead siRNA candidates was evaluated in in vitro infection models of SARS-CoV-1, SARS-CoV-2, HCoV-OC43, HCoV-NL63 and MERS-CoV. For all models except MERS-CoV, cells were reversely transfected with siRNAs at a final concentration of 10 nM using Lipofectamine RNAiMAX (1 µL per well; Thermo Fisher Scientific, Germany) in 24 well plates. siRNA-lipid complexes were prepared in 100 µL Opti-MEM I (Gibco, Thermo Fisher Scientific, Germany) and added to each well, followed by 500 µL of cell suspension. A549^hACE2^ cells were used for SARS-CoV-1 and SARS-CoV-2 assays (120,000 cells/well in complete DMEM), BHK-21 cells for HCoV-OC43 (30,000 cells/well in DMEM supplemented with 2% FBS), and Huh-7.5 cells for HCoV-NL63 (30,000 cells/well in DMEM supplemented with 2% FBS). Mock controls were included by treating cells with transfection reagent alone.

Cells were infected with the respective virus at an MOI of 0.1. For MERS-CoV experiments, Calu-3 cells were seeded on 24 well plates (540,000 cells/well in DMEM/F-12 supplemented with 10% FBS). After 24 h, cells were transfected with siRNAs at a final concentration of 25 nM using Oligofectamine reagent (2 µL per well; Invitrogen by Thermo Fisher Scientific, Germany). siRNA-lipid complexes were prepared in 50 µL Opti-MEM I (Gibco, Thermo Fisher Scientific, Germany) and added to the cells in 200 µL DMEM/F-12 without FBS after one washing step. 4 h post transfection, medium with FBS was added and cells were incubated for 48 h at 37 °C. Mock controls received transfection reagent alone. MERS-CoV infection was performed under Biosafety level 4 conditions at the Institute of Virology, Philipps University of Marburg, Germany as described before [50].

In experiments involving HCoV-OC43, HCoV-NL63, SARS-CoV-1 or SARS-CoV-2, cells were infected 4 to 6 h after siRNA transfection, whereas MERS-CoV infection was performed 48 h after siRNA transfection. HCoV-OC43 and HCoV-NL63 infections were carried out at 35 °C, and SARS-CoV-1, SARS-CoV-2 and MERS-CoV infections at 37 °C. Cell lysates for RT-qPCR analysis were harvested 24 h (MERS-CoV), 48 h (HCoV-OC43, SARS-CoV-1, SARS-CoV-2), or 96 h (HCoV-NL63) after infection.

### Quantification of viral RNA by RT-qPCR

Viral RNA was extracted from cell lysates using the RNeasy^®^ Mini Kit (Qiagen GmbH, Hilden, Germany) for MERS-CoV experiments or the NucleoSpin^®^ RNA Mini kit (Macherey-Nagel, Dueren, Germany) for experiments involving the other CoVs. HCoV-OC43 and HCoV-NL63 RNA was quantified using a TaqMan™ Fast Virus 1-Step Master Mix (Applied Biosystems, Warrington, UK) on a ViiA 7 Real-Time PCR System (Life Technologies, Foster City, CA, USA). For SARS-CoV-1 and SARS-CoV-2, cDNA was synthesized with the Superscript™ III First-Strand Synthesis System (Thermo Fisher Scientific; Dreieich, Germany) and quantified on a LightCycler^®^ 480 with 480 SYBR Green I Master Mix (Roche Holding AG; Basel, Switzerland) using 18 S rRNA as a reference gene. MERS-CoV RNA was quantified using Luna^®^ Universal qPCR Master Mix by NEB (New England Biolabs, Ipswich, USA) following cDNA synthesis by the LunaScript RT SuperMix Kit (New England Biolabs, Ipswich, USA). Cycling conditions, primers and probes are provided in the Additional file 1, Tables S1 and S2.

### Statistical analysis

Statistical analyses were performed using GraphPad Prism v10.4.1. Data are presented as mean ± standard deviation (SD) from independent biological replicates with the number of replicates (n) indicated in the respective figure legends. When required, data were tested for normality using the Shapiro-Wilk test. Multiple group comparisons in luciferase reporter assays (Fig. 5B) were performed using unpaired two-tailed t-tests with Welch’s correction, followed by Holm-Šidák correction for multiple comparisons (α = 0.05). For antiviral efficacy assays against wild-type HCoVs (Fig. 7A), statistical differences were calculated using Brown-Forsythe and Welch ANOVA followed by Dunnett’s T3 multiple comparisons test. For experiments involving MERS-CoV (Fig. 7C, D), statistical differences were determined using unpaired two-tailed t-tests with Welch’s correction. Significance thresholds were: *p < 0.05, **p < 0.01, ***p < 0.001, ****p < 0.0001. p values ≥ 0.05 are denoted as non-significant (ns).

### AI-assisted technologies in the writing process

During the preparation of this work the authors used ChatGPT, a language model developed by OpenAI, exclusively to assist with editing, language refinement, and clarity of scientific writing. After using this tool, the authors reviewed and edited the content as needed and take full responsibility for the content of the publication.

## Results

### Generation of a database of full-length *Orthocoronavirinae* genome sequences

**Fig. 1:**
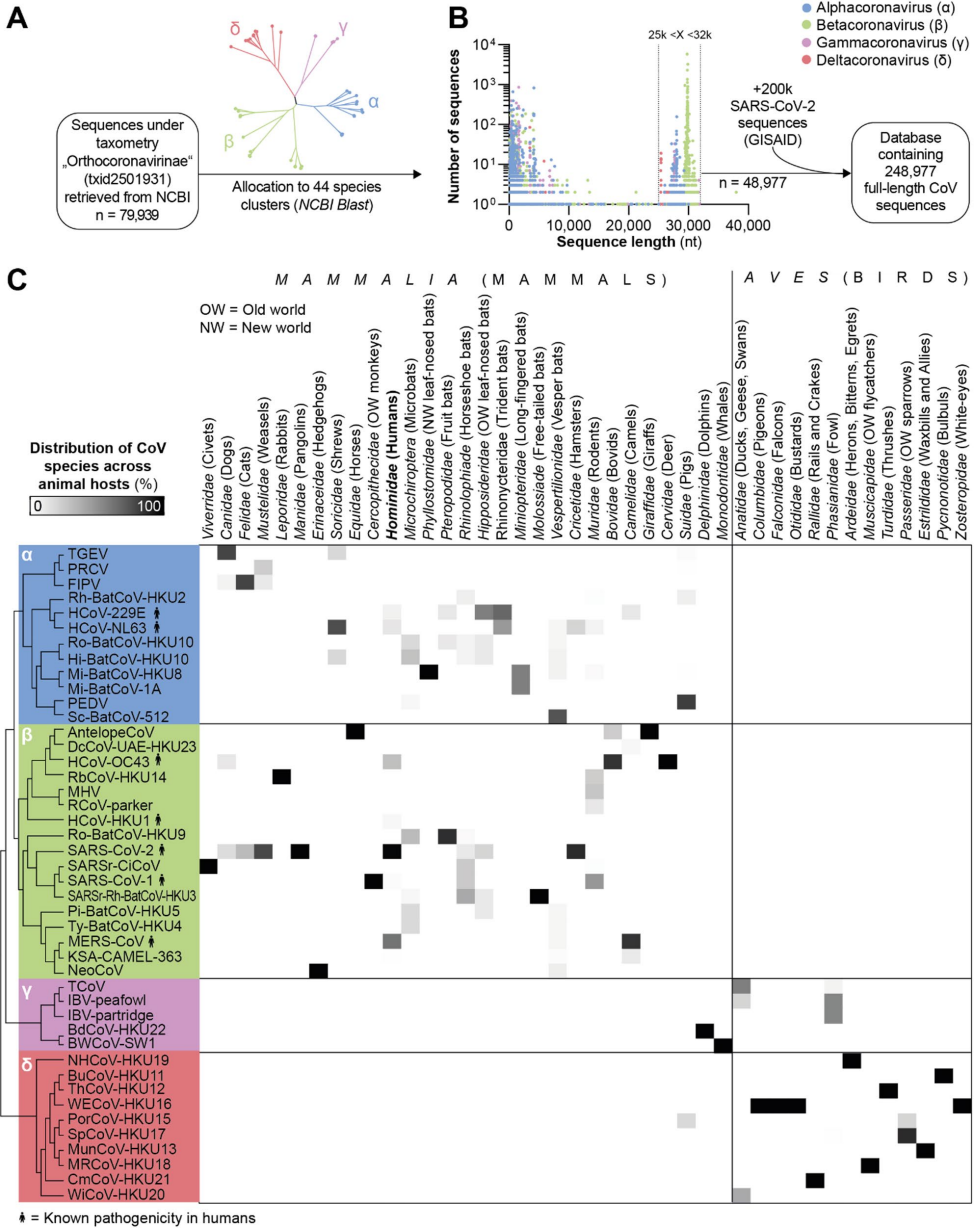
Generation of a database containing full-length *Orthocoronavirinae* genome sequences. **A***Orthocoronavirinae* genome sequences were retrieved from the National Center for Biotechnology Information (NCBI) Nucleotide Database including metadata. Sequences were allocated to species clusters by blasting against a set of 44 reference strains (further details in Additional file 1: Fig. S1). **B** Sequences with a length between 25,000 and 32,000 nucleotides were included for further analysis and an additional 200,000 SARS-CoV-2 sequences (including sequences of variants Alpha, Beta, Delta, Gamma, Omicron, Lambda and Mu) were added from the Global Initiative on Sharing All Influenza Data (GISAID) database. **C** Relative distribution of CoV species within each host family. The color intensity represents the proportion of isolates from a certain host that were assigned to the respective CoV species. The phylogenetic tree on the left represents the evolutionary relationship of CoV species, with the underlying color and Greek letters representing the CoV genus

To identify potential siRNA target sites that are conserved in different members of the *Orthocoronavirinae* subfamily, we constructed a custom, quality-filtered database of full-length CoV genome sequences [45]. From the National Center for Biotechnology Information (NCBI) Nucleotide Database, we retrieved 79,939 sequences classified under `*Orthocoronavirinae*´ (*n* = 79,939) without further restrictions. Sequences were allocated into species clusters (Fig. 1A) using the NCBI Basic Local Alignment Search Tool (BLAST) and a set of reference sequences that represented the different CoV species (Additional file 1: Fig. S1). This BLAST-based clustering was performed to harmonize species assignments across heterogeneous NCBI annotations. For 97.5% of sequences, BLAST and NCBI species assignments were identical, whereas the remaining 2.5% reflected limitations of NCBI taxonomy, including lower taxonomic resolution, host-based naming, legacy or non-standard nomenclature, or provisional classifications at the time of submission (Additional file 1: Fig. S2). Sequences shorter than 25 kilobases (kb) or longer than 32 kb were excluded to retain only complete genomes (Fig. 1B), as this size range reflects the expected intact genomic architecture of CoVs [2]. This length-based filtering did not formally guarantee the presence of all individual coding sequences, but substantially reduced the inclusion of fragmented or partial genomes. We finally included 48,977 sequences representing 44 distinct CoV species. An additional 200,000 SARS-CoV-2 sequences (including sequences of relevant variants of interest or concern as defined by the World Health Organization) were retrieved from the Global Initiative on Sharing All Influenza Data (GISAID) repository and integrated into the dataset. The resulting database represented CoVs that had been isolated from a wide range of hosts, spanning diverse mammalian species such as bats, primates, rodents, camels, cats, whales, or birds (Fig. 1C). Of note, CoVs isolated from mammalian hosts were exclusively classified within the *Alpha-* and *Betacoronavirus* genera, while gamma- and deltacoronaviruses were primarily found in birds. Exceptions included two gammacoronaviruses, which were derived from common bottlenose dolphins (BdCoV-HKU22) and beluga whales (BWCoV-SW1), as well as the deltacoronavirus PorCoV-HKU15, which was isolated from pigs.

### Conservation of potential siRNA target sites in the *Orthocoronavirinae* subfamily

**Fig. 2:**
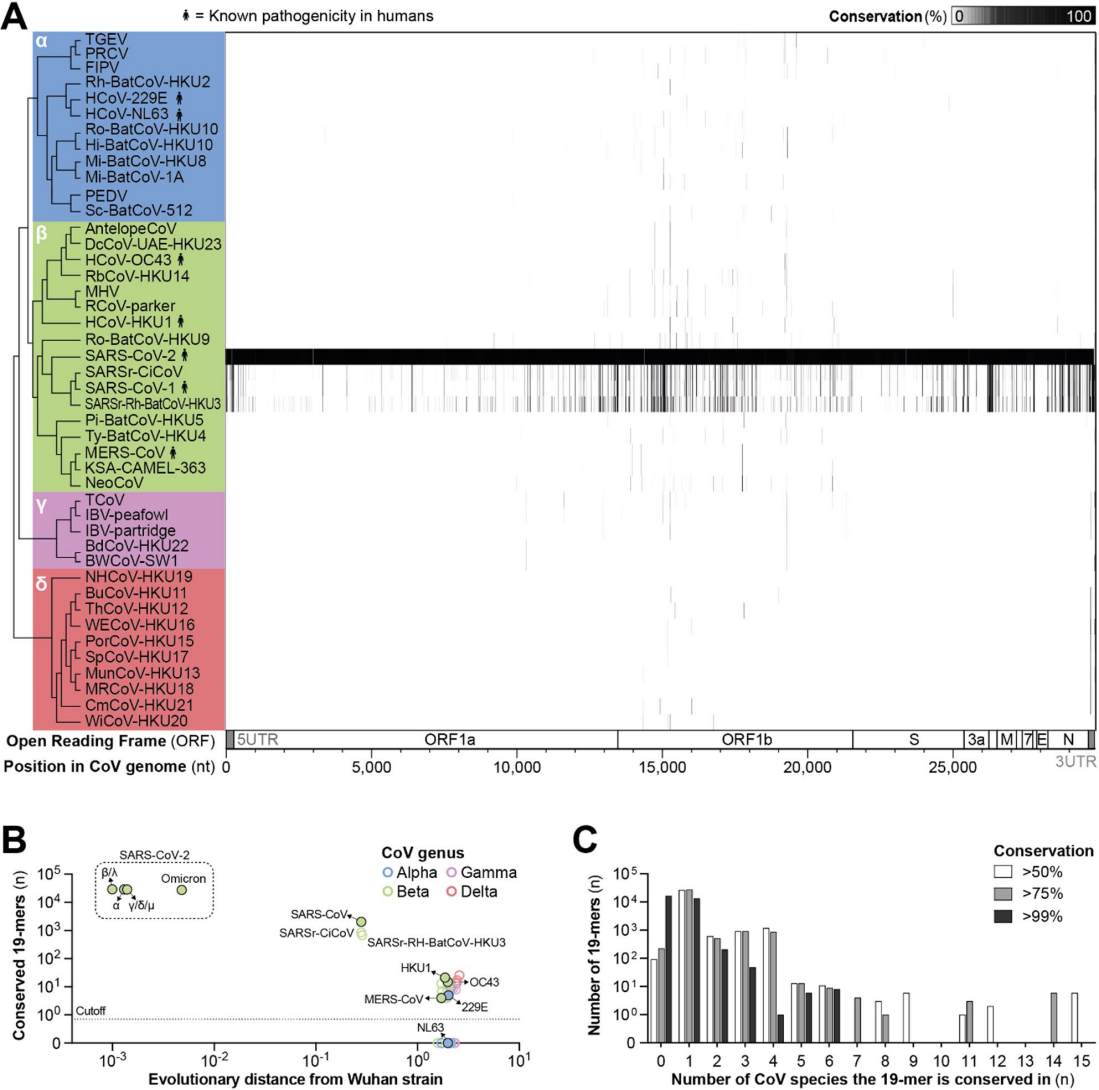
Conservation of 19-mer sequences within the genomes of *Orthocoronavirinae* species. **A** All potential 19-mer sequences (*n* = 29,884) were extracted from the SARS-CoV-2 Wuhan strain and analyzed for conservation within 44 *Orthocoronavirinae* (CoV) species, allowing a single mismatch at either position 1 or 19. The phylogenetic relationships of CoV species are shown on the left. Greek letters and underlying colors indicate the CoV genus. **B** Correlation of the number of 19-mer sequences that are conserved for at least 90% within SARS-CoV-2 variants (shown within the box) or other CoV species, and the evolutionary distance to the SARS-CoV-2 Wuhan strain. Filled circles with a black outline indicate HCoVs. **C** Distribution of 19-mer sequences according to the number of CoV species in which they are conserved

Using the SARS-CoV-2 Wuhan sequence as template, we extracted all possible 19-mer sequences (*n* = 29,871), corresponding to the minimal length of a typical siRNA target [51], and analyzed the conservation of each 19-mer within the CoV sequences in our database [45]. Conservation was defined as the proportion of sequences within a given CoV species or SARS-CoV-2 variant that contained the respective 19-mer, allowing a single mismatch at either position 1 or 19, as such mismatches are generally considered to have a negligible impact on siRNA activity [52]. While the majority of 19-mer sequences were conserved to a high degree within the SARS-CoV-2 population, this was not the case for other CoV species (Fig. 2A).

To better understand how the evolutionary relationship among CoV species affected the conservation of potential siRNA target sites, we correlated the number of highly conserved target sites (defined as 90% sequence conservation or more) within a specific CoV species with its evolutionary distance from the SARS-CoV-2 Wuhan strain (Fig. 2B). Overall, we found an inverse correlation, with a greater evolutionary distance associated with fewer conserved target sites. In line with this, the majority of analyzed 19-mer sequences (at least 28,121 out of 29,871, corresponding to 94%) were conserved across the different SARS-CoV-2 variants. The closest relatives of SARS-CoV-2, namely the other three sarbecoviruses SARS-CoV-1, SARSr-Rh-BatCoV-HKU3, and SARSr-CiCoV, still contained 690 to 2044 highly conserved 19-mer sequences. In contrast, the number of conserved sites decreased substantially in CoV species outside the *Sarbecovirus* subgenus. Even other members of the *Betacoronavirus* genus contained no more than 21 conserved 19-mer sequences, similar to the numbers observed in alpha-, delta-, and gammacoronaviruses. This reduction again correlated with the increased evolutionary distance, which was almost as high for these betacoronaviruses outside of the *Sarbecovirus* subgenus as for CoVs from other genera.

We now analyzed in how many different CoV species each 19-mer sequence was conserved (Fig. 2C). Notably, no single 19-mer was conserved across all species of the *Betacoronavirus* genus, let alone the whole *Orthocoronavirinae* subfamily. Even under the assumption of a relatively low conservation threshold of 50%, the most broadly conserved 19-mer sequences were identified in only 15 out of the 44 analyzed CoV species. This number dropped to 14 species at a 75% conservation threshold and declined further to just six species at 99% conservation (Fig. 2C). Importantly, the locations of conserved genomic sites differed widely across CoV genera. The majority of the 19-mers that were conserved across the *Alpha*- and *Betacoronavirus* genera were mapped to the ORF1b region, whereas 19-mers that were conserved in *Gamma*- and *Deltacoronavirus* species were primarily located in the 3’ untranslated region (UTR) (Fig. 2A).

### Selection of siRNA candidates for screening of antiviral activity

**Fig. 3:**
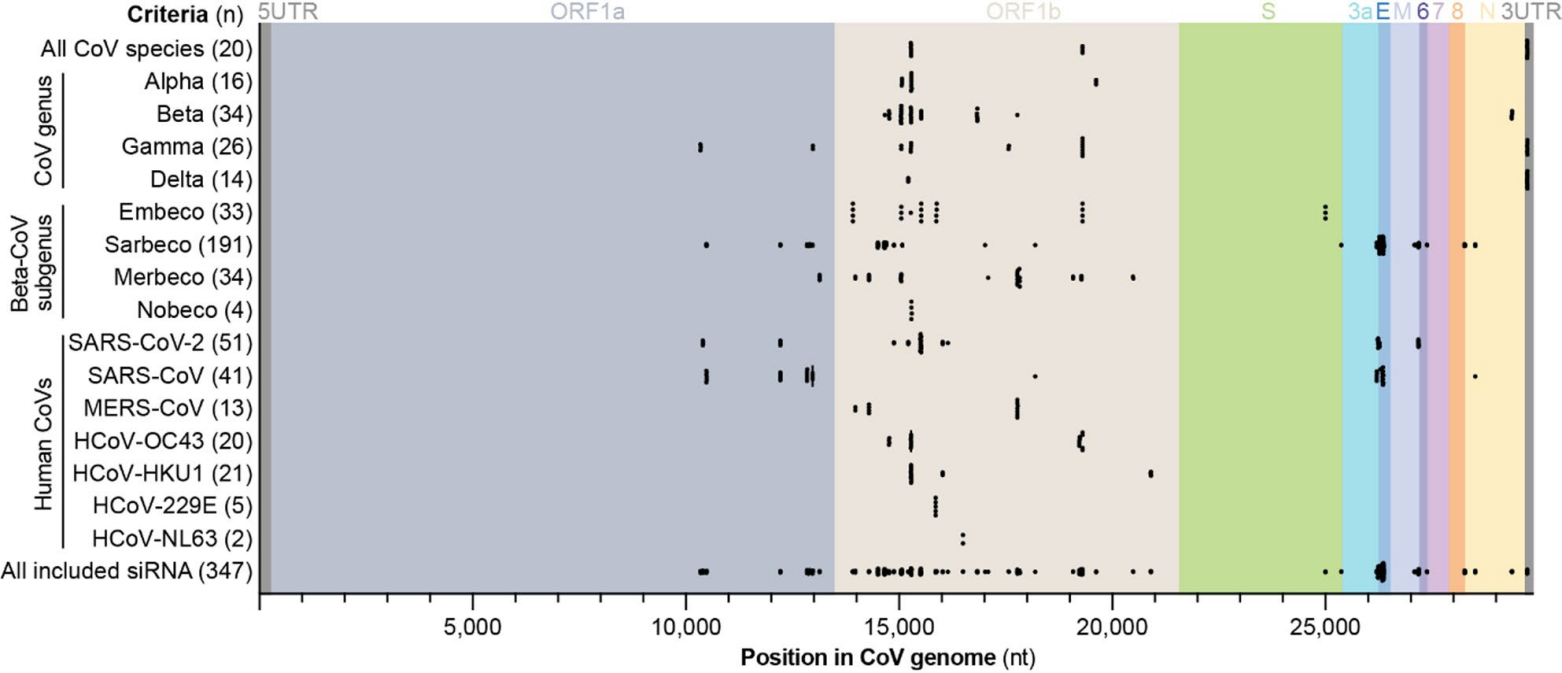
Criteria for selecting siRNA candidates and distribution of siRNAs across CoV genomes. **A** Selection criteria for target sites are shown on the left, with the number of siRNAs selected for each criterion indicated in brackets. The siRNAs with the highest mean conservation within each criterion were selected. The open reading frames (ORFs) of the SARS-CoV-2 genome are labeled on top (UTR = untranslated region, S = spike glycoprotein, E = envelope protein, M = membrane protein, N = nucleocapsid protein). The color code for ORFs was adopted from Kim et al. *Cell* 2020 [53]

Based on the observation that most highly conserved 19-mer sequences were shared across different, only partially overlapping groups of CoV species, we implemented a multi-layered strategy to select siRNA candidates. Target sequences were included based on several criteria, such as demonstrating the highest mean conservation throughout all species of (i) the whole CoV subfamily, (ii) a certain CoV genus (*Alpha-*,* Beta-*,* Gamma-*,* Deltacoronavirus*), (ii) a certain *Betacoronavirus* subgenus (*Embecovirus*,* Sarbecovirus*,* Merbecovirus*,* Nobecovirus*), or (iii) among the seven HCoV species (Fig. 3).

The selected siRNAs targeted either the genome region between 10 and 21 kb (ORF1a/b) or 25–30 kb, including coding sequences of the spike glycoprotein (S), open reading frame (ORF) 3a, envelope protein (E), membrane protein (M), ORF6, ORF8, nucleocapsid protein (N), and the 3´UTR. In contrast, no 19-mer from the genomic regions between 0 and 10 kb or 21 to 25 kb met our selection criteria. 19-mers with highest mean conservation across all CoV species were found either in the ORF1b or the 3´UTR, while the majority of chosen target sites were located in the ORF1b region. Target sites in ORF1a were primarily chosen for their conservation within the *Gammacoronavirus* genus or the *Sarbecovirus* subgenus. On the other hand, 19-mers within the S, ORF3a, E, M, ORF6, ORF8 or N genes were selected for their conservation within the *Embeco*- or *Sarbecovirus* subgenera. Lastly, additional target sites located within the 3´UTR were selected due to their overlap with delta- and gammacoronaviruses. Several of the 19-mers fulfilled multiple conservation criteria, ultimately leading to the selection of 347 distinct target sites.

### Screening antiviral activity and tolerability of siRNA candidates in a high-throughput SARS-CoV-2 infection model

**Fig. 4:**
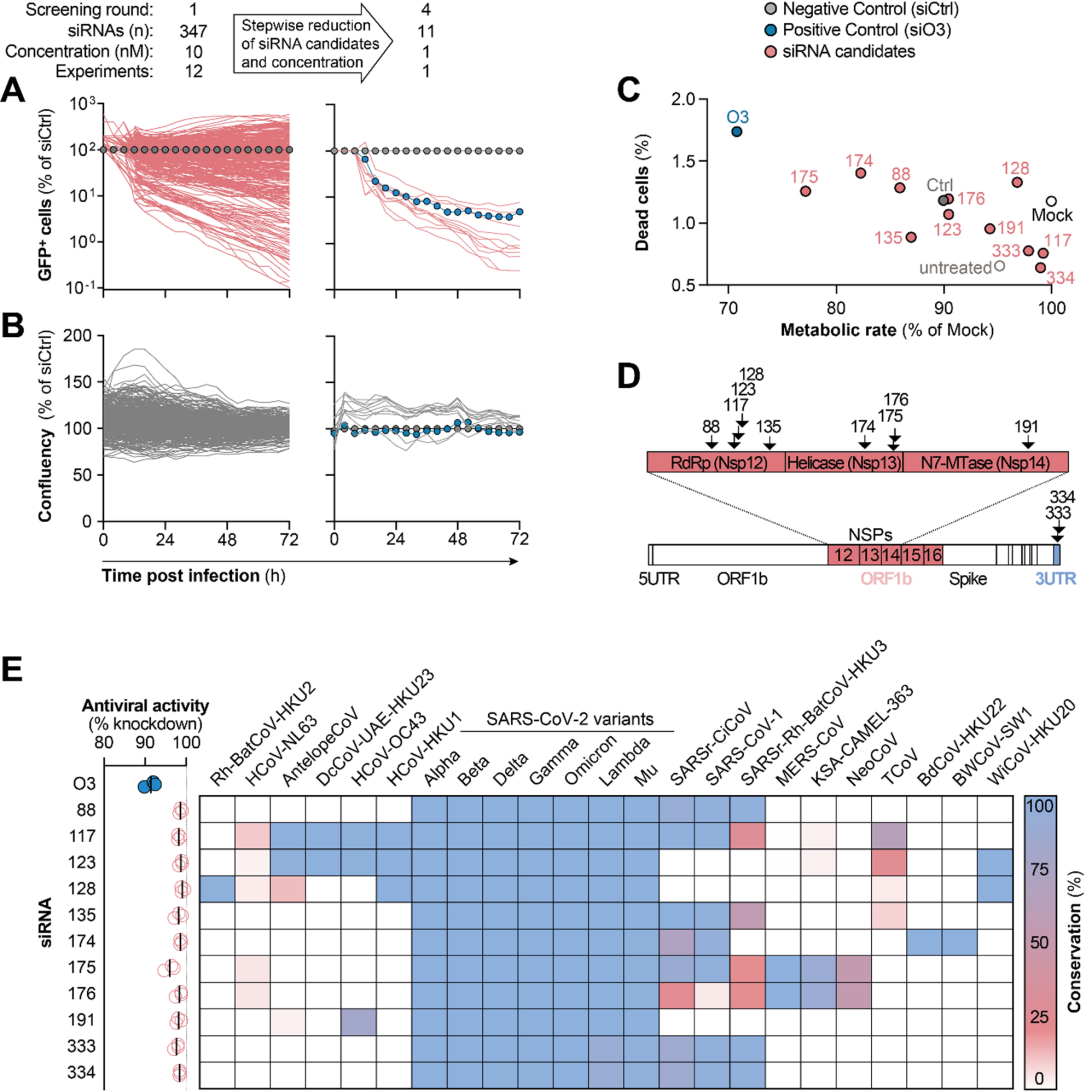
Screening of antiviral activity in a SARS-CoV-2 infection model. **A** To screen for antiviral activity, A549 cells overexpressing the human angiotensin converting enzyme 2 were transfected with siRNAs at a concentration of 1 to 10 nM and infected 6 h later with a recombinant SARS-CoV-2 (rSARS-CoV-2^GFP^) expressing the green fluorescent protein (GFP). The number of GFP^+^ cells and (**B**) cell confluency were quantified by time-lapse fluorescence microscopy. **C** Phenotypic screening for signs of toxicity of eleven selected siRNAs at a concentration of 10 nM by quantifying the percentage of dead cells relative to total cells per well and the metabolic rate of cells 72 h post transfection. **D** Location of target sites in the CoV genome of eleven selected siRNAs. **E** Conservation of siRNA target sites. Only CoV species in which a conservation of more than 10% was observed for an siRNA candidate are shown. Graph on left shows knockdown of rSARS-CoV2^GFP^ 72 h post infection (same experimental setup as in panel A)

We designed 347 siRNAs against the selected target sites using the SARS-CoV-2 Wuhan strain as a template and assessed antiviral activity in a high-throughput SARS-CoV-2 infection model. This model employed a recombinant SARS-CoV-2 strain that expressed the green fluorescent protein (GFP) from a transgene replacing the viral ORF7 gene (rSARS-CoV-2^GFP^) [54]. GFP^+^ cells as a surrogate marker for viral infection were quantified by time-lapse fluorescence microscopy [32]. Six hours after siRNA transfection, A549 cells overexpressing the human angiotensin converting enzyme 2 (A549^hACE2^) were infected with rSARS-CoV-2^GFP^, and viral spread (Fig. 4A) as well as cell confluency (Fig. 4B) were monitored over three days. Multiple screening rounds were conducted, each time employing increasingly stringent selection criteria while progressively reducing the siRNA concentration. Candidates were chosen for their superior antiviral activity, tolerability or target site conservation. Eleven of the most promising siRNA candidates were evaluated in greater detail for potential toxicities. This included the in silico prediction of RNAi off-target activity against the human transcriptome (Additional file 1: Fig. S3), as well as phenotypic assays to quantify dead cells and metabolic activity following siRNA transfection (Fig. 4C). Five of the eleven siRNAs (si88, si117, si123, si128, si135) targeted the coding sequence of the RNA-dependent RNA polymerase (RdRp), three targeted the helicase (si174, si175, si176), one (si191) targeted the guanine-N7-methyltransferase (N7-MTase), and two targeted the 3’UTR of the viral genome (si333, si334) (Fig. 4D). All eleven siRNAs demonstrated superior safety profiles (Fig. 4C) and enhanced antiviral activities (Fig. 4E) when compared to the lead siRNA from our previous study [32], siO3.

### Optimization of siRNA design and chemistry enhances potency

**Fig. 5:**
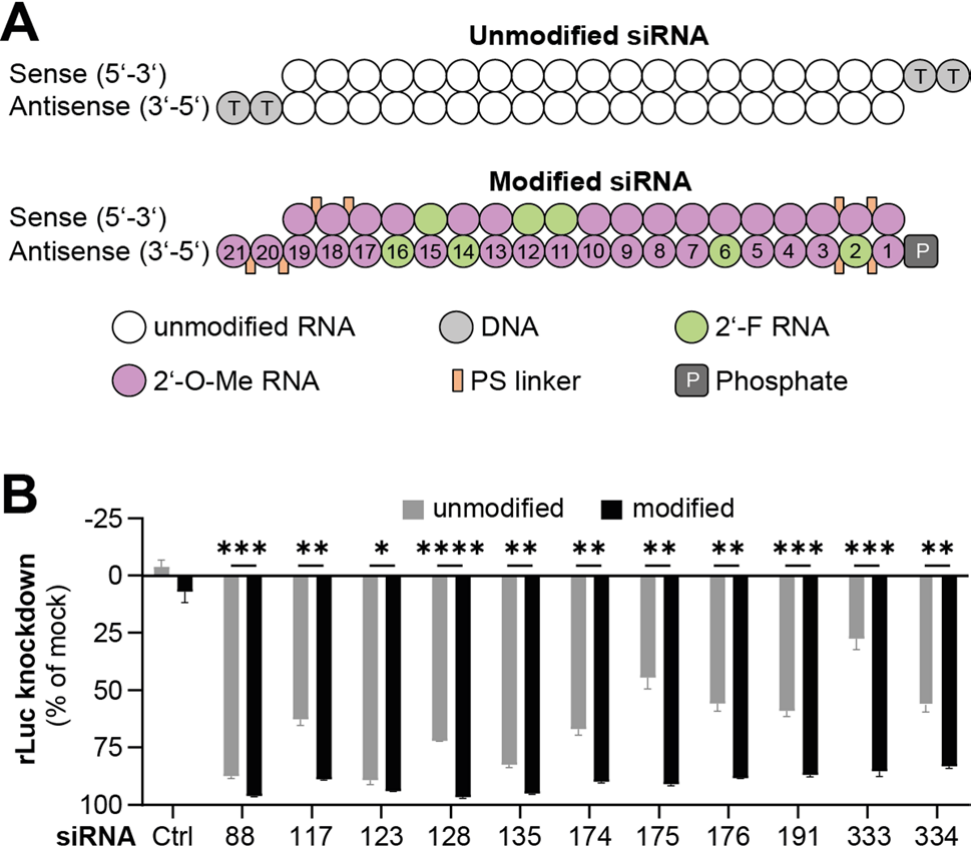
Chemical modifications enhance siRNA activity. **A** Design and modification pattern of unmodified siRNA with dTdT overhang (top) and modified siRNA incorporating 2’-O-methyl (2’-O-Me), 2′-fluoro (2′-F) and phosphorothioate (PS) linkage modifications (bottom). **B** Silencing efficiencies of unmodified (gray bars) and chemically modified (black bars) siRNA candidates were compared using dual-luciferase reporters. siRNAs were co-transfected at 0.1 nM with a reporter containing the SARS-CoV-2 siRNA target site in the 3´UTR of *Renilla* luciferase (rLuc). Bars represent mean ± SD (*n* = 4). Statistical significance was assessed by unpaired two-tailed t-tests with Welch’s correction and Holm–Šidák adjustment for multiple comparisons (α = 0.05): *p < 0.05, **p < 0.01, ***p < 0.001, ****p < 0.0001

The siRNAs used for the high-throughput screening were designed as standard 19-mer duplexes with dTdT overhangs at the 3´ ends of both strands (Fig. 5A). To enhance stability and minimize immunogenicity, a siRNA design and chemical modification pattern similar to clinically approved siRNA-based drugs [55–57] was employed. Specifically, 2’-fluoro (F) and 2’-O-methyl (O-Me) ribose modifications were incorporated in a defined pattern [58], along with two terminal 3´-phosphorothioate (PS) linkages at both ends of each strand (Fig. 5A). The reference siRNA design is furthermore asymmetric, typically comprising a 23 nt antisense strand and a 21 nt sense strand, with a 2 nt overhang at the 3´ end of the antisense strand [59]. As our conservation analysis was based on 19-mer sequences (Fig. 2), both siRNA strands were shortened by 2 nt to obtain a 19 nt sense strand and a 21 nt antisense strand (Fig. 5B). In line with the reference siRNA design [58], the 2 nt 3’ overhang of the antisense strand was adjusted to match the target site sequence derived from the Wuhan SARS-CoV-2 strain.

The introduction of the modified siRNA design and chemical modifications significantly enhanced the silencing activity of all tested siRNA candidates (Fig. 5B). All eleven modified siRNAs demonstrated potent suppression of luciferase reporters carrying fully complementary target sites, achieving knockdown efficiencies of more than 80% at a concentration of 0.1 nM, with only minor differences observed between the most active candidates.

### Cross-reactivity of siRNA candidates against target site variations found in HCoVs

After having observed efficient silencing of luciferase reporters containing perfectly matched target sites derived from SARS-CoV-2, the cross-reactivity of siRNA candidates against the other HCoVs was investigated.

**Fig. 6:**
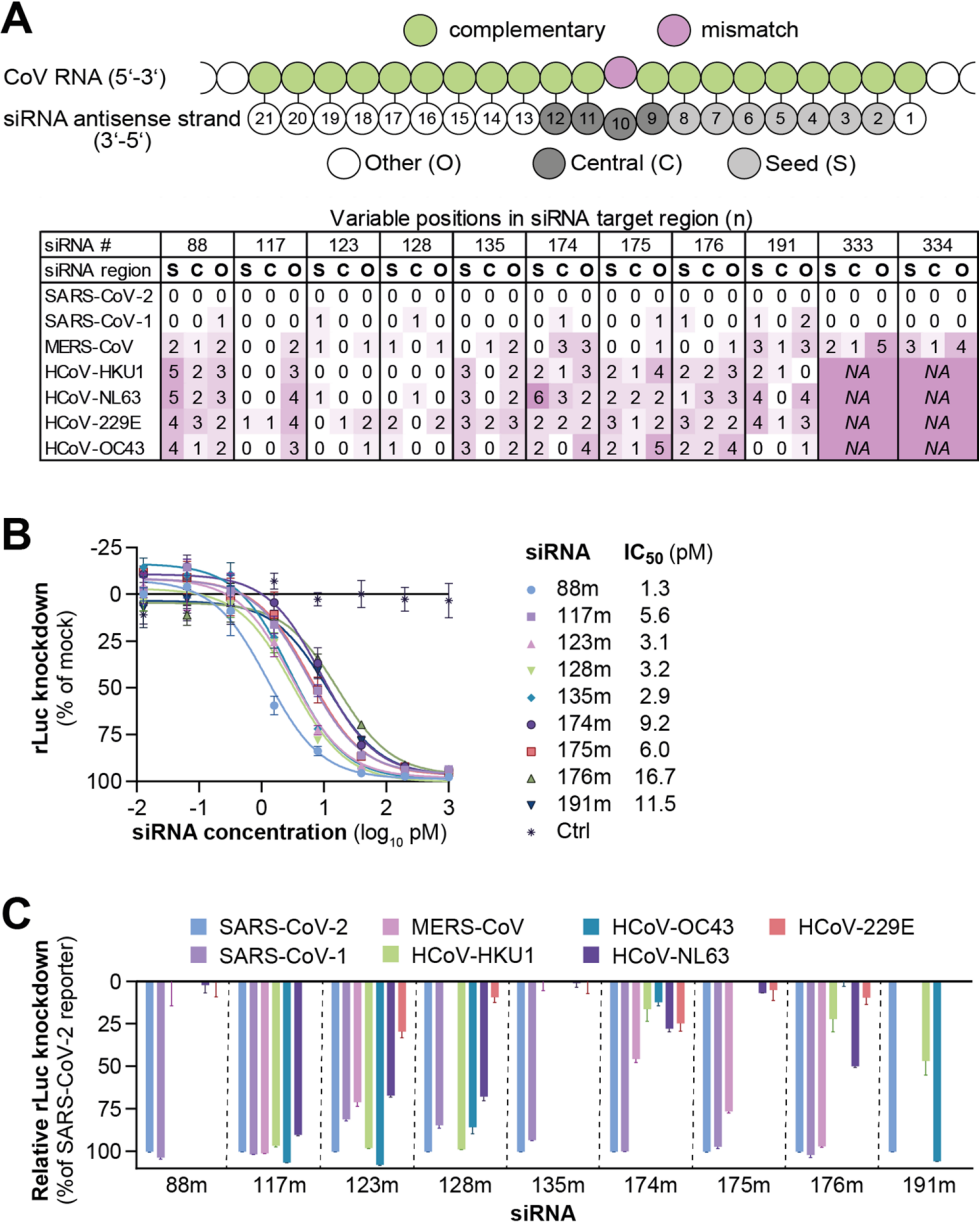
Mismatch analysis and cross-reactivity of siRNA against target sites of HCoVs. **A** Conservation of siRNA target sites within HCoV species. A position was classified as variable if less than 98% of the analyzed sequences contained the nucleotide complementary to the siRNA sequence. The scheme at the top depicts the definition of siRNA regions used to categorize variable positions within the target sites. S = seed region (positions 2 to 8 of the antisense strand); C = central region (positions 9 to 12 of the antisense strand); O = other (positions 1 and 13 to 21 of the antisense strand). The table summarizes the number and regional distribution of variable positions within each siRNA target site for each HCoV species (bottom). *NA* (not applicable) indicates that no homologous target sequence could be identified in the respective HCoV species. **B** Half-maximal inhibitory concentrations (IC_50_) of chemically modified siRNAs (siRNAm) were determined using a dual-luciferase reporter containing the fully complementary (SARS-CoV-2-derived) target sites in the 3´UTR of *Renilla* luciferase (rLuc). **C** Evaluation of siRNA activity against the different HCoV target site variations using dual-luciferase reporters. siRNAs were co-transfected at a dose representing 100 x IC_50_ values, along with plasmids containing the respective variation of the siRNA target site in the 3´UTR of rLuc. rLuc knockdown values are shown relative to mock-treated controls and were normalized to the corresponding SARS-CoV-2 reporter. Data represent mean ± SD (*n* = 3 to 6). Full graph also depicting negative values is provided in Additional file 1: Fig. S9

Given that mismatches between the siRNA and the viral target can reduce silencing activity [52], we analyzed the conservation of each nucleotide position of the siRNA target sites across the seven HCoV species (Additional file 1: Fig. S1 4 and S5). Since we had adapted the 2 nt overhangs at the 3´end of the antisense strand to the Wuhan strain (Fig. 5A), positions 20 and 21 were also included in the analysis. The positions of the target sites were grouped according to the functional regions of the siRNA antisense strand (Fig. 6A), as mismatches in the seed region (positions 2 to 8) or central region (positions 9 to 12) are known to impact siRNA activity more profoundly [52, 60], compared to mismatches at position 1 or positions 13 to 21 [61]. A position was classified as variable if less than 98% of the analyzed sequences contained the nucleotide complementary to the siRNA sequence. The conservation of individual nucleotide positions was generally high across SARS-CoV-2 variants with an average conservation of 99.91% and a minimum conservation of 99.09% (Additional file 1: Fig. S4 and S5). Notably, the lowest conservation was observed at position 19 of si117 and could be attributed to a mutation that was exclusively found in the Alpha variant of SARS-CoV-2 (Additional file 1: Fig. S6 and S7), which is no longer circulating. Most siRNAs showed also a high degree of conservation across SARS-CoV-1 sequences, with target sites containing either no (si117, si135, si333, si334) or only one (si88, si123, si128, si174, si175, si176) variable position, whereas si191 contained three variable positions (Fig. 2B). In contrast, the number of variable positions varied substantially across siRNA candidates for the other five HCoVs. Notably, the target sites of si333 and si334, located in the 3´UTR of the viral genome, were entirely absent in HCoV-HKU1, HCoV-229E, HCoV-OC43 and HCoV-NL63, leading to the exclusion of si333 and si334 from further analyses. The target sites of si88, si135, si174, si175, si176, and si191 likewise contained a substantial number of variable positions in several HCoVs, which also included the seed and central region of the siRNA. In contrast, the target sites of si117, si123, and si128, all located within the RdRp, demonstrated the highest degree of conservation. Notably, si117 exhibited variable positions in the seed or central region exclusively within HCoV-229E sequences, suggesting broad compatibility with the other six HCoVs (Fig. 6A).

To compare the potency of the nine remaining siRNA candidates in more detail, the half-maximal inhibitory concentrations (IC_50_) of the modified siRNAs (siRNAm) were determined using luciferase reporters with fully complementary target sequences (Fig. 6B). si88m, si135m, si123m, si128m and si117m demonstrated the highest activity with exceptionally low IC_50_ values ranging from 1.3 pM (si88m) to 5.6 pM (si117m). In contrast, the highest IC_50_ values were measured for si191m (11.5 pM) and si176m (16.7 pM).

We next examined the impact of mismatches within the siRNA target sites on silencing activity. To this end, the target sites derived from reference sequences of the seven HCoVs (Additional file 1: Fig. S1) were cloned into luciferase reporters, and silencing by siRNAs was investigated (Fig. 6C). The luciferase knockdown by siRNAs correlated with both the number and position of mismatches (Additional file 1: Fig. S8), with mismatches in the seed or central region having a stronger negative effect than mismatches at other locations. For instance, si135m completely lost activity against the MERS-CoV reporter, which contained a single mismatch in the central region, combined with two additional mismatches at positions 15 and 21 of the siRNA target site. In contrast, two mismatches outside the seed or central region alone had no substantial effect on knockdown efficiency against the same reporter for si117m. As expected, the three siRNAs si117m, si123m and si128m with the lowest target site variability achieved the most comprehensive knockdown of HCoV reporters. si117m demonstrated the broadest spectrum of activity, efficiently suppressing the target sequences of SARS-CoV-2, SARS-CoV-1, MERS-CoV, HCoV-HKU1, HCoV-OC43, and HCoV-NL63 (Fig. 6C). Notably, si117m did not achieve knockdown of the HCoV-229E reporter, consistent with the presence of mismatches in both the seed and central regions of the 229E target site.

In summary, the siRNA candidates varied substantially in their degree of sequence conservation across HCoV genomes, resulting in marked differences in their spectrum of activity. Three siRNAs targeting the RdRp coding region, si117m, si123m, and si128m, demonstrated high cross-reactivity against sequences derived from multiple HCoVs.

### Lead candidate si117m exhibits broad antiviral activity against multiple HCoVs

The antiviral activity of the three most promising siRNA candidates si117m, si123m and si128m was evaluated using multiple wild-type HCoVs, including two SARS-CoV-2 strains (EU1 and XBB1.15), SARS-CoV-1, HCoV-OC43, HCoV-NL63 and MERS-CoV. The siRNAs were transfected into susceptible cell lines and viral replication was evaluated by quantifying viral gRNA and total viral RNA from cell lysates using reverse transcription quantitative PCR (RT-qPCR). For MERS-CoV, 50% tissue culture infectious doses (TCID_50_) were additionally determined, as this virus causes a more pronounced cytopathic effects than the other evaluated HCoVs.

**Fig. 7:**
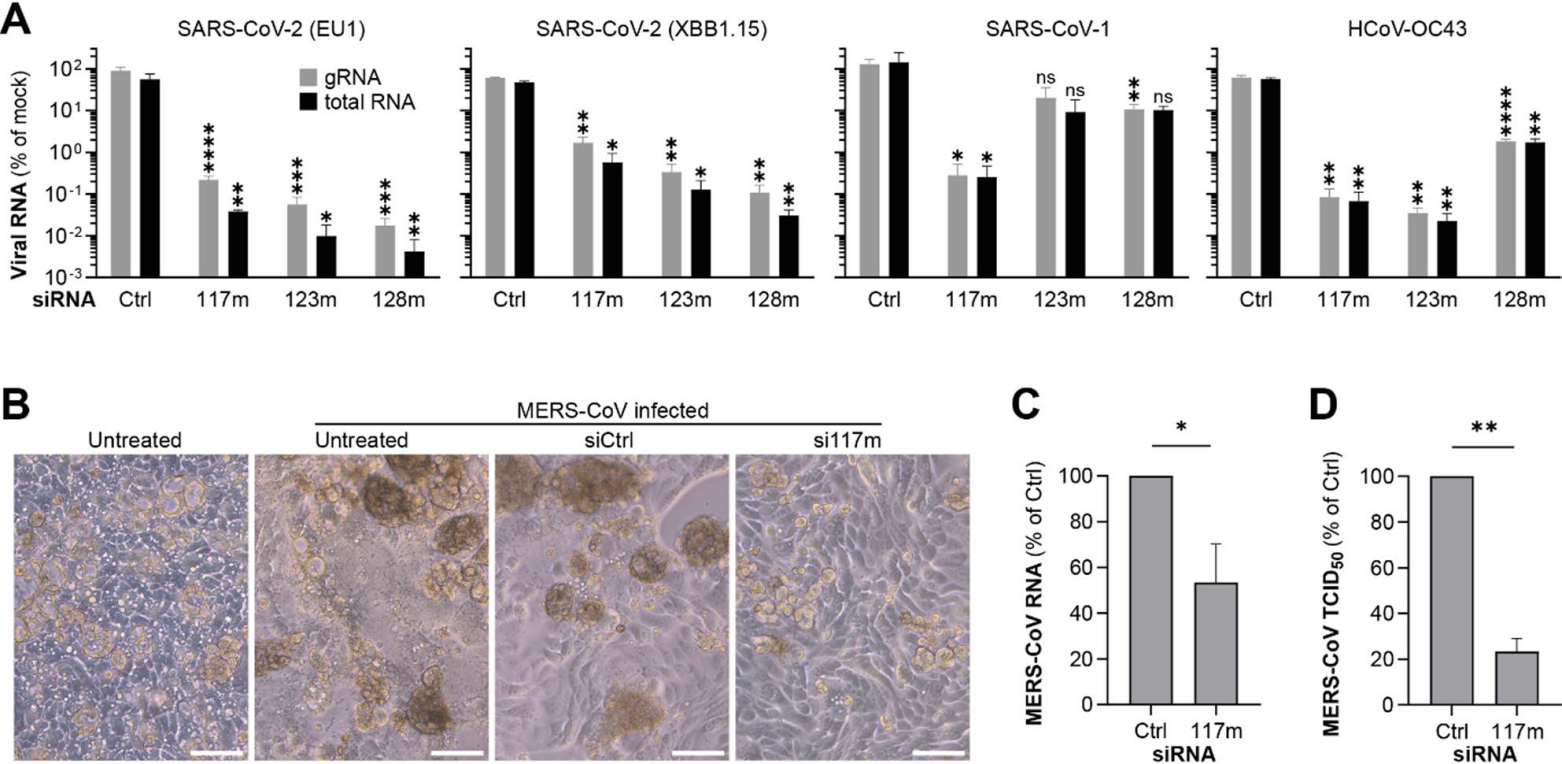
Antiviral activity of siRNA candidates against representative wild-type HCoVs. **A** The antiviral activity of chemically modified siRNA candidates was evaluated against SARS-CoV-2 (EU1 and XBB.1.15 variants), SARS-CoV-1, and HCoV-OC43. A549 cells overexpressing the human angiotensin converting enzyme 2 were used for experiments involving SARS-CoV-1 and SARS-CoV-2, and BHK-21 cells for the experiment with HCoV-OC43. Cells were transfected with siRNAs at a dose of 10 nM using Lipofectamine RNAiMAX 4 h before viral infection (MOI = 0.1). Viral replication was assessed 48 h after infection by quantifying viral total or genomic RNA (gRNA) in cell lysates using reverse-transcription quantitative PCR. Bars represent mean ± SD (*n* = 3). Statistical significance was assessed using Brown–Forsythe and Welch ANOVA followed by Dunnett’s T3 multiple comparisons test to compare each siRNA to the non-targeting control siRNA (Ctrl). **B**-**D** Antiviral activity of si117m against MERS-CoV. Calu-3 cells were transfected with si117m (25 nM) using Oligofectamine as transfection reagent and 48 h later infected with MERS-CoV (MOI = 0.1). **B** Cytopathic effects were assessed by bright-field microscopy 24 h post infection. The scale bar at bottom right represents 100 μm. Viral replication was assessed 24 h post infection by quantifying (**C**) viral RNA from cell lysates using reverse-transcriptase quantitative PCR, and (**D**) infectious virions from supernatants by determining the 50% tissue culture infectious doses (TCID₅₀). Statistical significance was calculated using unpaired two-tailed t-tests with Welch’s correction. ns = non-significant, *p < 0.05, **p < 0.01, ***p < 0.001, ****p < 0.0001

In general, the three siRNAs exhibited strong antiviral activity against SARS-CoV-2 and HCoV-OC43, suppressing viral RNA levels by up to 5 log_10_, whereas antiviral effects against SARS-CoV-1 were primarily observed for si117m (Fig. 7A). No substantial differences were observed between the suppression of viral total or gRNA. Consistent with our initial screening results using rSARS-CoV-2^GFP^ (Fig. 4E), si128m demonstrated the strongest suppression of SARS-CoV-2 replication for both analyzed strains. At a 10 nM concentration, si128m reduced total viral RNA of the EU1 variant by 4.6 log_10_, compared to a 4.0 log_10_ reduction by si123m or 3.6 log_10_ by si117m. A similar trend was observed for the XBB.1.15 variant, although the knockdown of viral RNA was on average 1 log_10_ lower, potentially due to an intrinsically lower replication efficiency [62, 63].

For the closely related SARS-CoV-1, si117m exhibited the strongest antiviral efficacy, consistent with the luciferase reporter results (Fig. 6C), reducing total viral RNA levels by 2.7 log_10_. In contrast, si123m and si128m showed reductions of approximately 1 log_10_, which reached statistical significance only for viral gRNA in the case of si128m. A potential explanation could be that the SARS-CoV-1 target regions carried a mismatch in the seed region for si123m and in the central region for si128m (see Fig. 6A). For HCoV-OC43, si117m and si123m reduced total viral RNA levels by 3.3 log₁₀ and 3.8 log₁₀, respectively, indicating strong antiviral activity. In contrast, si128m achieved only a 1.8 log_10_ reduction, again reflecting mismatches in the seed region of the siRNA.

While all CoVs investigated so far belonged to the *Betacoronavirus* genus, we now evaluated whether our siRNAs could also suppress the alphacoronavirus HCoV-NL63. In contrast to our previous findings, none of the three siRNA candidates significantly inhibited HCoV-NL63 replication (Additional file 1: Fig. S10). This result was expected for si123m and si128m due to mismatches in the critical seed and central regions. The lack of antiviral activity of si117m, however, was less expected. Although the target site of si117m carried three mismatches, all of these were located at the 3´ end of the antisense strand at positions 19 to 21, which are typically less important for silencing. Nonetheless, this finding also reflected the luciferase reporter results, in which si117m achieved a weaker knockdown of the HCoV-NL63 reporter compared to the other HCoV reporters (Fig. 6C).

Given its broad antiviral activity and favorable safety profile (Fig. 4), si117m was selected for further evaluation in a MERS-CoV infection model. Infection of Calu-3 cells with MERS-CoV induced extensive cytopathic effects, which were markedly reduced in si117m-treated cells (Fig. 7B). Consistent with these observations, si117m treatment significantly inhibited MERS-CoV replication, reducing intracellular viral RNA levels by 47% (Fig. 7C) and the number of infectious virions in supernatant by more than 77% compared to the control siRNA (Fig. 7D). In summary, si117m demonstrated cross-species antiviral activity against multiple HCoVs, including SARS-CoV-2, SARS-CoV-1, HCoV-OC43 and MERS-CoV.

## Discussion

CoVs are a global health threat, with seven species known to cause pathology in humans. While antiviral drugs are available to treat COVID-19, no approved therapies are available for diseases caused by the other six HCoVs, let alone by potential future pandemic strains. The development of antivirals with broad activity against multiple CoV species is therefore a critical priority for the global research community.

While we and others have previously demonstrated that individual CoV species can be effectively targeted by siRNAs [29, 32, 35–39], no siRNA with activity against multiple CoV species has been reported so far. Also, it remained unclear whether the high genetic variability of CoVs would allow the development of such broadly active siRNAs. To address this question, we performed a comprehensive analysis of *Orthocoronavirinae* genomics to identify conserved target sites, and screened the antiviral activity of nearly 350 siRNA candidates in a SARS-CoV-2 infection model. Further refinement using virus-specific luciferase reporters and wild-type HCoVs led to the identification of si117m, a chemically modified siRNA that efficiently suppressed replication of SARS-CoV-1, SARS-CoV-2, MERS-CoV, and HCoV-OC43. While we were not able to test the antiviral activity against HCoV-HKU1 due to its poor replication in cell culture models [64], our data using luciferase reporters indicate that si117m might also be active against this HCoV.

Beyond identifying broadly active siRNA candidates, our study provides insights into the evolutionary diversity and host distribution of CoVs. The phylogenetic analysis we performed confirmed that alpha- and betacoronaviruses are restricted to mammalian hosts, while gamma- and deltacoronaviruses primarily infect avian species. The only exceptions are the gammacoronaviruses BdCoV-HKU22 and BWCoV-SW1, which affect dolphins and whales, respectively, as well as the delta-CoV PorCoV-HKU15, which circulates in pigs. These findings align with previous reports suggesting that interspecies transmission and recombination events may facilitate avian-to-mammalian spillover [65].

As expected, the conservation of siRNA target sites correlated with the evolutionary distance of CoV species from the SARS-CoV-2 Wuhan strain. Notably, we found a relatively low target site conservation among betacoronaviruses outside the *Sarbecovirus* subgenus. This finding reflected the substantial genetic diversity within the *Betacoronavirus* genus, in which the evolutionary distance between sarbecoviruses and other betacoronaviruses was similar as the distance between sarbecoviruses and members of the *Alpha-*,* Gamma-*, or *Deltacoronavirus* genera. The remarkably high genetic diversity within the *Orthocoronavirinae* subfamily appears particularly striking when compared to that of mammals. For instance, the evolutionary distance between humans and pigs was estimated at 0.2-0.3 [66], approximately in the same range as the distance between SARS-CoV-2 and the other *Sarbecovirus* species SARS-CoV-1, SARSr-RH-BatCoVHKU3 and SARSr-CiCoV. In contrast, the evolutionary distance between SARS-CoV-2 and HCoV-OC43 was determined as 2.0, which is six to ten times higher than the distance between humans and pigs. This extensive diversity underscores the fundamental challenge of designing antiviral therapies that are effective across multiple HCoVs. Consistent with this, at least 690 siRNA target sites were conserved for more than 90% within members of the *Sarbecovirus* subgenus, whereas no single site was substantially conserved across all CoVs.

Notably, si117m and the other two top-performing candidates si123m and si128m target the genomic region that encodes for the RdRp catalytic center, a region known to be structurally and functionally conserved across CoVs [67]. Targeting such a functionally constrained domain may contribute to the broad antiviral activity observed for these siRNAs, as mutations in this region are less likely to be tolerated without compromising viral fitness. In addition, targeting ORF1 supports our previous findings that siRNAs directed against the ORF1 region offer a strategic advantage by avoiding the highly abundant subgenomic RNAs [32]. Although a single siRNA effective against all CoVs could not be identified, our lead candidate si117m achieved broad coverage among HCoVs of highest clinical relevance, such as all three pandemic HCoVs. A further expansion of the antiviral spectrum may be achieved by leveraging siRNA cocktails. Combining multiple siRNAs may not only broaden the spectrum of activity but could also increase the resistance barrier, if needed [13, 14].

During our analysis, we defined the siRNA target site as a 19-mer but allowed one mismatch at either position 1 or 19, effectively capturing sequences with 18 conserved nucleotides. Another potential strategy to identify siRNAs with broader activity could be to shorten the target site during conservation analysis, thereby increasing the chances of identifying sequences that are conserved across additional CoV species. We had initially explored this more relaxed approach by allowing mismatches at positions 1, 18, and 19 of the 19-mer, effectively analyzing the conservation of 16-mer sequences. However, this strategy did not yield substantially more target sites (data not shown), while creating the risk of reduced silencing efficacy, which is why we chose to allow only a single mismatch at position 1 or 19. Thus, our approach of screening 19-mers with one mismatch allowed either at positions 1 or 19 balanced the need for a high target site conservation with the practical requirements of effective siRNA design [51].

A potential limitation of our study could be that we used the SARS-CoV-2 Wuhan strain as a template to define the siRNA target sites. We cannot exclude that a potential 19-mer, which was not found in the reference sequence of the Wuhan strain and therefore was not considered in our analysis, might be conserved in an even higher number of CoV species. Nonetheless, given the relevance of SARS-CoV-2 as a human pathogen, neglecting such sequences reflects a clinically motivated design decision to prioritize viruses that pose the highest threat to humans.

A crucial strength of our study was the screening of antiviral activity of siRNA candidates using a fully replication competent SARS-CoV-2 reporter virus. The automated quantification of infected cells by time-lapse fluorescence microscopy enabled the testing of a relatively large number of siRNA candidates in an authentic infection model. It also ensured that only siRNAs with genuine antiviral activity were selected for further evaluation, which would not have been achieved through a reporter-based screening alone. Regarding HCoV-NL63, all lead siRNAs produced measurable knockdown in luciferase assays but failed to suppress replication in the infection model, highlighting the limitations of reporter systems. This aligns with the findings by Hariharan et al. [38], who demonstrated that luciferase reporter assays often overestimate antiviral efficacy. One potential explanation is that mismatch tolerance differs between assay systems. Notably, si117m, which carries three mismatches at positions 19 to 21, exhibited partial knockdown of the HCoV-NL63 reporter but completely lost activity in the corresponding infection model. This indicates that even mismatches at the 3’ end, which are generally considered to be well tolerated [52, 60, 68], can critically impair silencing efficacy under physiological conditions. Additional factors, such as a reduced target accessibility due to RNA secondary structures or the shielding by viral nucleocapsid proteins, as well as the dynamics of viral replication [38, 69], may be further explanations for this discrepancy. Moreover, the functional role of viral gRNA differs from that of standard mRNA. While both reporter mRNA transcripts and viral RNAs can be subject to cleavage or translational repression, viral gRNA also serves as a replication template [70]. Consequently, cleavage of viral gRNA may eradicate the virus from an infected cell, whereas translational repression without cleavage may still permit genome replication and transcription at later stages, thereby limiting the antiviral effect. This functional difference may contribute to the loss of efficacy observed in infection models, even when reporter systems appear to be not affected. These findings underscore the limitations of applying general mismatch rules across all target contexts and emphasize the importance of validating antiviral siRNA candidates in infection models rather than relying solely on reporter assays.

The chemical modification pattern applied to our siRNAs is based on clinically validated designs, enhancing nuclease resistance and minimizing immunogenicity [58]. These modifications likely contributed to the high potency of our siRNAs and could translate to long dosing intervals especially when used as a prophylactic. Supporting this, siRNAs with similar designs have shown in vivo protection against SARS-CoV-2 for up to four weeks after a single dose [14], underscoring the potential of long-acting RNAi therapeutics.

Effective delivery to the respiratory tract will be a key next step for clinical translation of our findings. Approaches such as inhaled formulations [71, 72] using lipid nanoparticles [73] or alternative carriers [28] are currently under investigation. The recent success of ARO-RAGE, an inhalable, peptide-conjugated siRNA in development for the treatment of inflammatory pulmonary diseases, which achieved target knockdown for over 3 months in a phase 1/2a clinical trial [31, 74], further highlights the feasibility of durable RNAi-based therapies for respiratory diseases.

## Conclusions

Our study presents a comprehensive analysis of siRNA target site conservation across the *Orthocoronavirinae* subfamily. We demonstrate the high genetic variability among CoVs and the corresponding challenge of designing broadly active RNAi therapeutics. Despite the absence of a universally conserved target site, we were able to develop si117m, a chemically modified siRNA that potently inhibited replication of SARS-CoV-1, SARS-CoV-2, MERS-CoV, and HCoV-OC43, and expression of a HCoV-HKU1 reporter. Given its cross-species antiviral activity and its tolerability, si117m represents a promising candidate for clinical translation.

## Supplementary Information

Additional file 1. This file contains the supplementary figures S1 – S10 and supplementary tables S1 and S2.

## Data Availability

All sequence datasets, alignments, and analysis scripts supporting the findings of this study are publicly available via Zenodo under DOI [https://doi.org/10.5281/zenodo.18471403](https:/doi.org/10.5281/zenodo.18471403) . Any additional information required to reanalyze the data reported in this study is available from the lead contact upon request. Unique and stable reagents generated in this study are available from the lead contact with a completed materials transfer agreement.

## Abbreviations

siRNA: small interfering RNA
RNAi: RNA interference
CoV: Coronavirus
HCoVs: Human coronavirus
nt: nucleotide
RISC: RNA-induced silencing complex
RSV: Respiratory syncytial virus
LNP: Lipid nanoparticle
BLAST: Basic Local Alignment Search Tool
kb: kilobase
NCBI: National Center for Biotechnology Information
GISAID: Global Initiative on Sharing All Influenza Data
S: Spike glycoprotein
N: Nucleocapsid protein
M: Membrane protein
E: Envelope protein
rSARS-CoV-2^GFP^: recombinant SARS-CoV-2 expressing GFP
A549h^ACE2^: A549 cells overexpressing the human angiotensin converting enzyme 2
N7-MTase: Guanine-N7-methyltransferase
F: 2’-fluoro RNA modification
O-Me: 2’-O-methyl RNA modification
PS: 3´-phosphorothioate linkage
COVID-19: Coronavirus disease 2019
ORF: Open reading frame
UTR: Untranslated region
GFP: Green fluorescent protein
RdRp: RNA-dependent RNA polymerase
IC_50_: Half-maximal inhibitory concentration
gRNA: Genomic RNA
TCID_50_: 50% tissue culture infectious dose
RT-qPCR: Reverse transcription quantitative PCR
SD: Standard deviation
