# Supplementary material for: Comparative genomics identifies small interfering RNA with activity against all five human betacoronaviruses
Source: Genome Med. 2026 Apr 22;18:44. doi: 10.1186/s13073-026-01659-1 (PMC13101182; doi:10.1186/s13073-026-01659-1)
Supplement: Supplementary file 1 — Additional file 1. This file contains the supplementary figures S1 – S10 and supplementary tables S1 and S2. [file 13073_2026_1659_MOESM1_ESM.docx]

# Supplementary data for manuscript:

**Comparative genomics identifies small interfering RNA with activity against all five human betacoronaviruses**


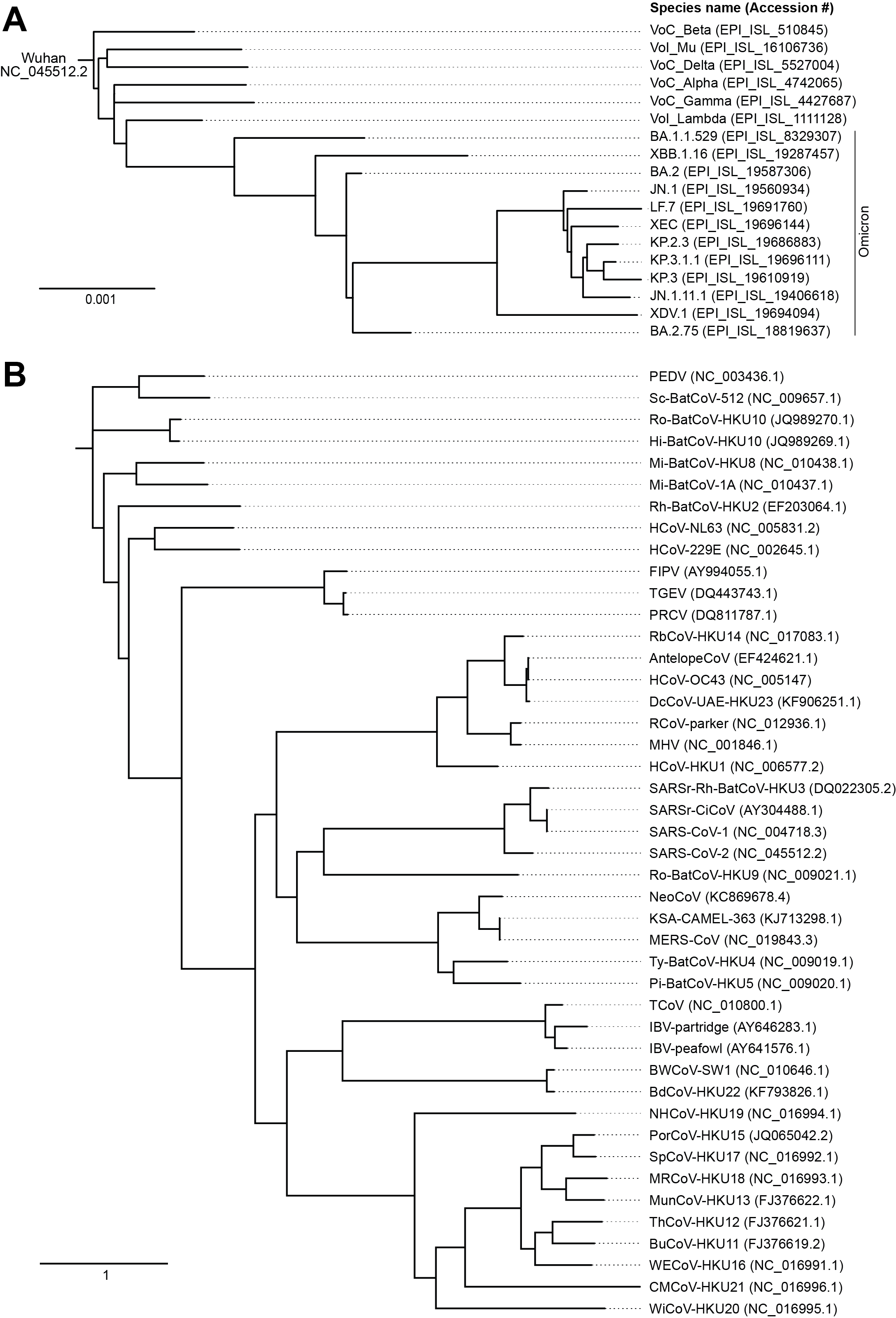


**Fig S1. Phylogenetic tree of reference sequences used to categorize Orthocoronavirinae sequences.** References used to categorize (A) SARS-CoV-2 strains into the specific variants or (B) allocate non-SARS-CoV-2 sequences to a specific species are shown. Accession numbers are shown in brackets. Bar at lower left of graphs represents evolutionary distance. Abbreviations: PEDV, porcine epidemic diarrhea virus; Sc-BatCoV 512, *Scotophilus* bat coronavirus 512; Ro-BatCoV HKU10, *Rousettus* bat coronavirus HKU10; Hi-BatCoV HKU10, *Hipposideros* bat coronavirus HKU10; Mi-BatCoV HKU8, *Miniopterus* bat coronavirus HKU8; Mi-BatCoV 1A, *Miniopterus* bat coronavirus 1A; Rh-BatCoV HKU2, *Rhinolophus* bat coronavirus HKU2; HCoV-NL63, human coronavirus NL63; HCoV-229E, human coronavirus 229E; FIPV, feline infectious peritonitis virus; TGEV, transmissible gastroenteritis virus; PRCV, porcine respiratory coronavirus; RbCoV HKU14, rabbit coronavirus HKU14; AntelopeCoV, sable antelope coronavirus; HCoV-OC43, human coronavirus OC43; DcCoV UAE-HKU23, dromedary camel coronavirus UAE-HKU23; RcoV parker, rat coronavirus Parker; MHV, murine hepatitis virus; HCoV-HKU1, human coronavirus HKU1; SARSr-Rh-BatCoV HKU3, SARS-related *Rhinolophus* bat coronavirus HKU3; SARSr-CiCoV, SARS-related palm civet coronavirus; SARS-CoV-1, SARS coronavirus 1; SARS-CoV-2, SARS coronavirus 2; Ro-BatCoV-HKU9, *Rousettus* bat coronavirusHKU9; NeoCoV, coronavirus *Neoromicia*/PML-PHE1/RSA/2011; KSA-CAMEL-363, KSA-CAMEL-363 isolate of Middle East respiratory syndrome coronavirus; MERS-CoV, Middle East respiratory syndrome coronavirus; Ty-BatCoV-HKU4, *Tylonycteris* bat coronavirus HKU4; Pi-BatCoV-HKU5, *Pipistrellus* bat coronavirus HKU5; TcoV, turkey coronavirus; IBV-partridge, avian infectious bronchitis virus partridge isolate; IBV-peafowl, avian infectious bronchitis virus peafowl isolate; BWCoV-SW1, beluga whale coronavirus SW1; BdCoV HKU22, bottlenose dolphin coronavirus HKU22; NHCoV HKU19, night heron coronavirus HKU19; PorCoV HKU15, porcine coronavirus HKU15; SpCoV HKU17, sparrow coronavirus HKU17; MRCoV HKU18, magpie robin coronavirus HKU18; MunCoV HKU13, munia coronavirus HKU13; ThCoV HKU12, thrush coronavirus HKU12; BuCoV HKU11, bulbul coronavirus HKU11; WECoV HKU16, white-eye coronavirus HKU16; CMCoV HKU21, common-moorhen coronavirus HKU21; WiCoV HKU20, wigeon coronavirus HKU20


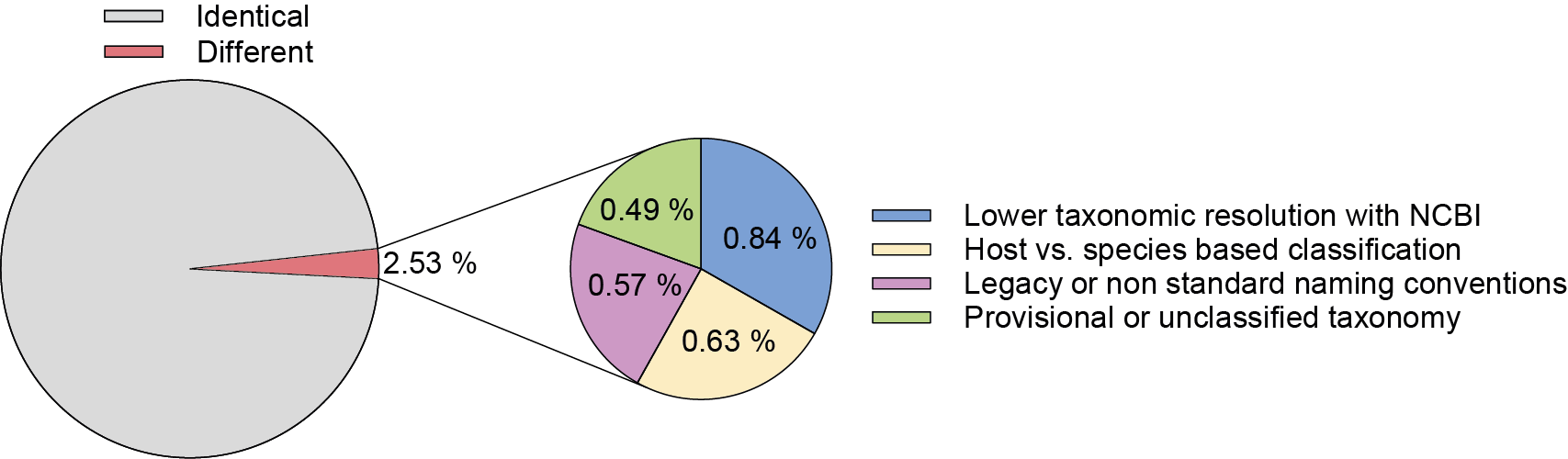


**Fig S2. Comparison of BLAST-based species assignments with NCBI taxonomy.**
Overview of concordance between BLAST-based species clustering and NCBI-provided taxonomy across all 48,977 CoV genomes analyzed. The left pie chart shows the proportion of sequences with identical species assignments (97.47%) versus differing assignments (2.53%). The right pie chart breaks down the non-identical cases into four explanatory categories: lower taxonomic resolution in NCBI annotations compared with the reference sequence set, host-based (NCBI) versus virus species-based (BLAST) naming, legacy or non-standard nomenclature in NCBI records, and provisional or unclassified taxonomy at the time of submission. Percentages in the right chart refer to the fraction of all sequences.


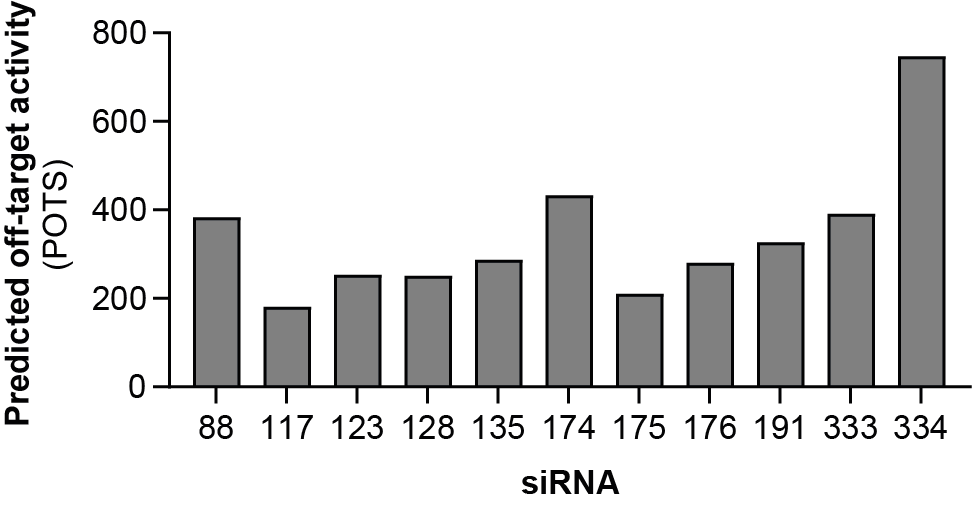


**Fig S3. Sequence based off-target prediction of eleven siRNA candidates.** Off-target potential was assessed using the siSPOTR algorithm, which calculates the Probability of Off-Targeting Score (POTS) based on complementarity of the siRNA seed region to the human transcriptome. Lower POTS values indicate reduced likelihood of off-target interactions


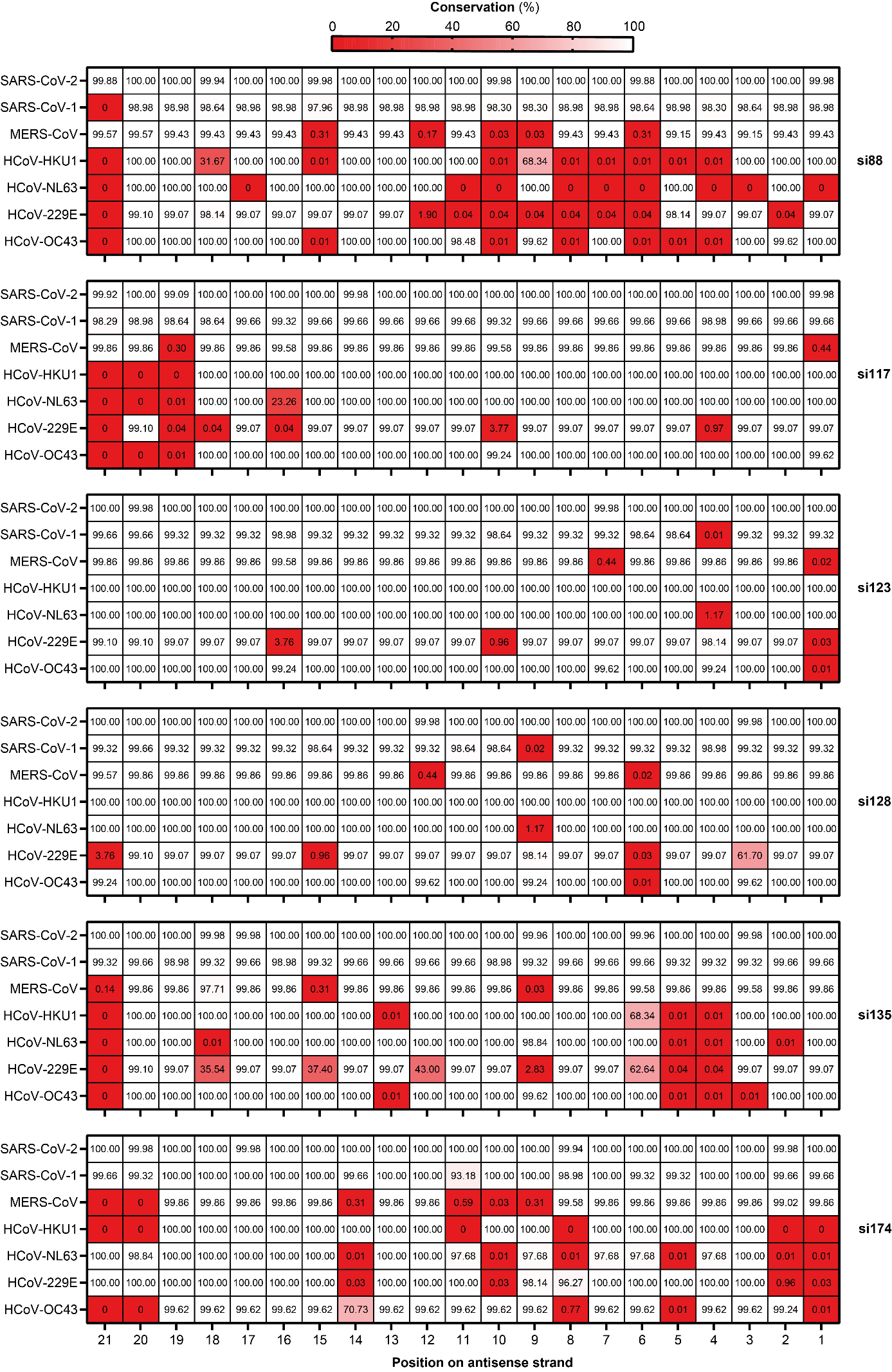


**Fig S4. Conservation of individual nucleotide positions of the target sites of si88, si117, si123, si128, si135, and si174 across HCoVs.** For all HCoVs except SARS-CoV-2, conservation was calculated from alignments of sequences that were contained in the database shown in Fig.1 and Fig. 2. For SARS-CoV-2, the conservation analysis was based on a globally representative multiple sequence alignment obtained from the Nextstrain SARS-CoV-2 open dataset. Conservation is expressed as the percentage of analyzed sequences within each CoV species that contain the complementary base to the siRNA antisense strand. The conservation is indicated by the color code and % values given in cells.


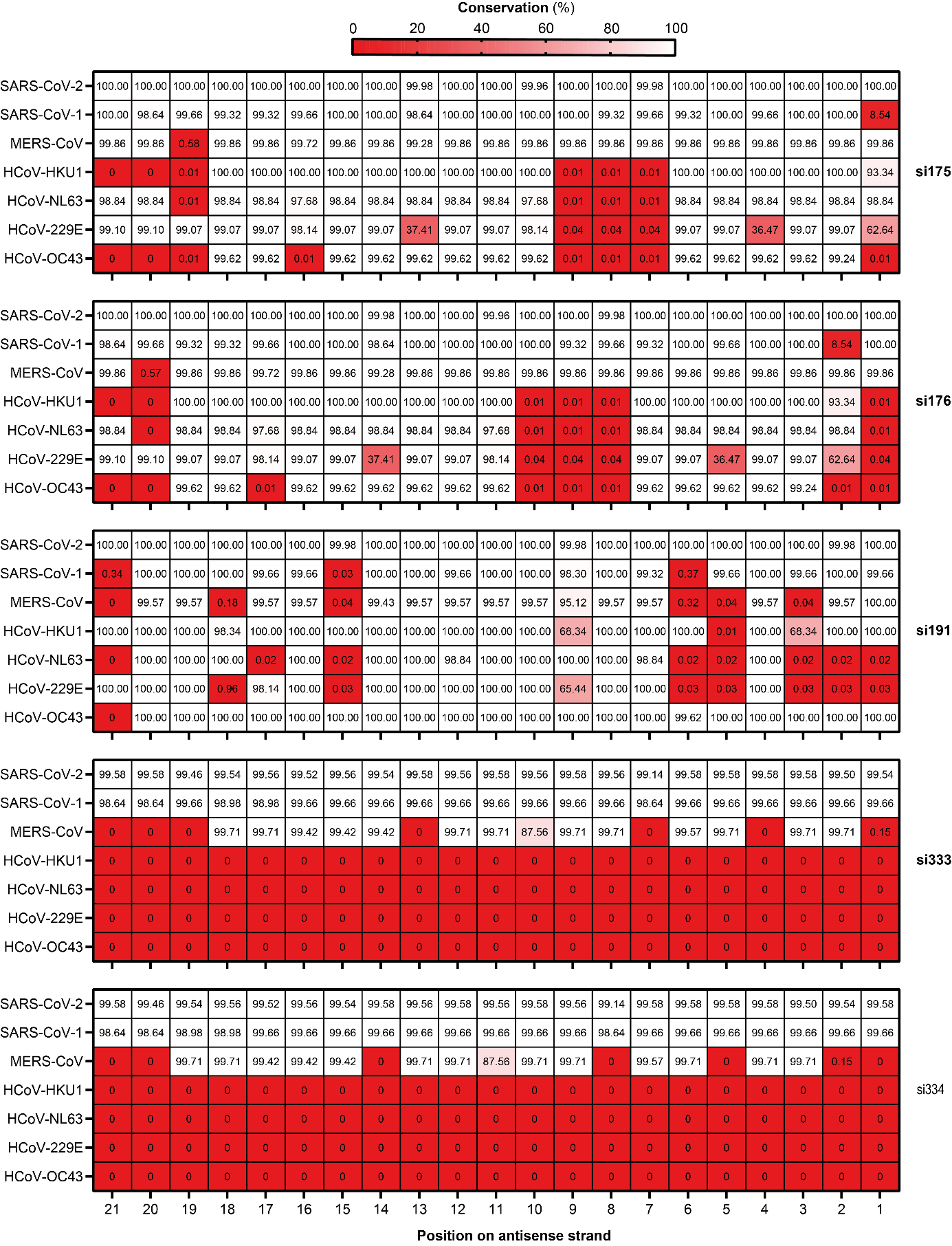


**Fig S5. Conservation of individual nucleotide positions of the target sites of si175, si176, si191, si333, and si334 across HCoVs.** For all HCoVs except SARS-CoV-2, conservation was calculated from alignments of sequences that were contained in the database shown in Fig.1 and Fig. 2. For SARS-CoV-2, the conservation analysis was based on a globally representative multiple sequence alignment obtained from the Nextstrain SARS-CoV-2 open dataset. Conservation is expressed as the percentage of analyzed sequences within each CoV species that contain the complementary base to the siRNA antisense strand. The conservation is indicated by the color code and % values given in cells.


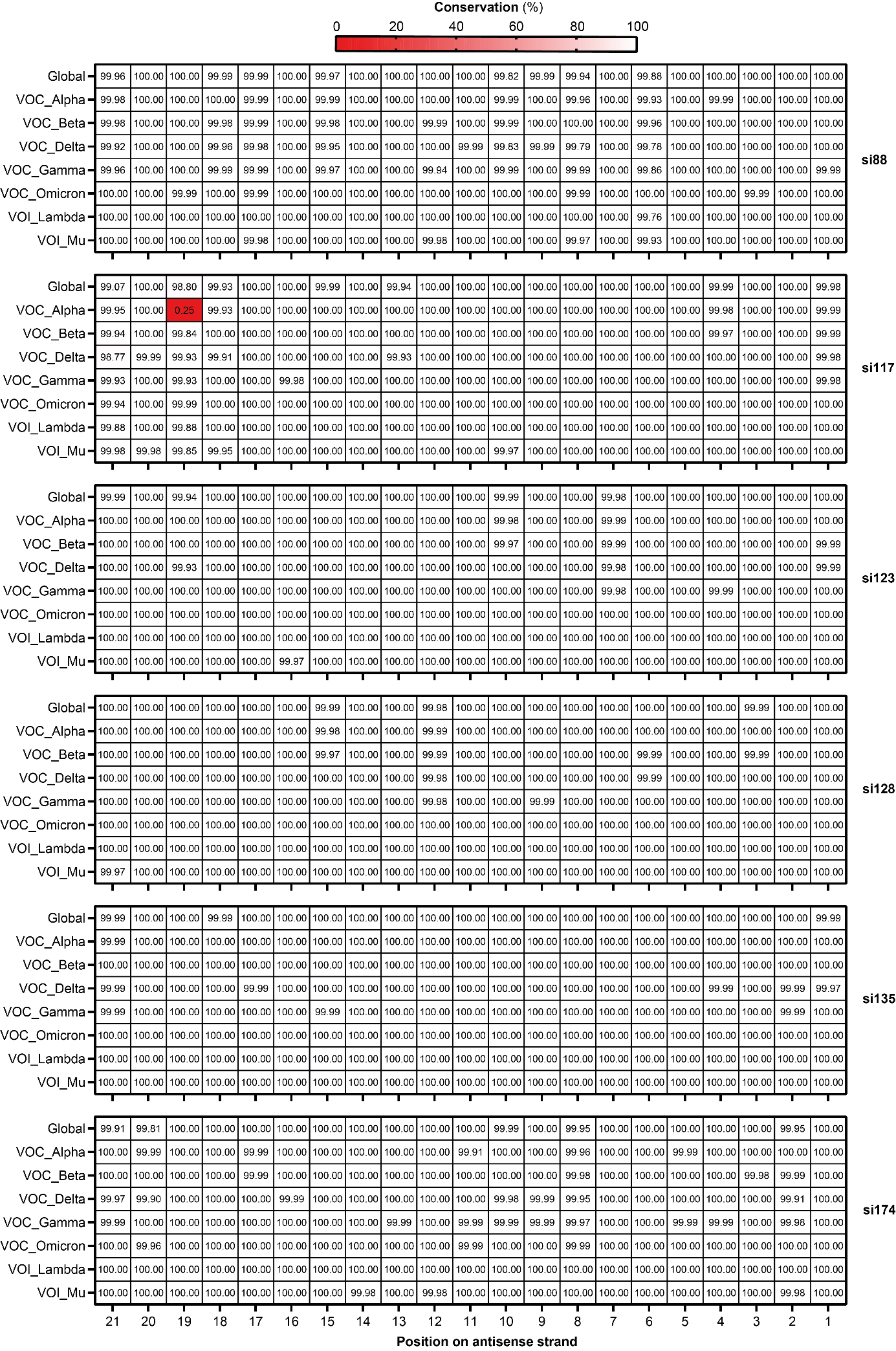


**Fig S6.** **Conservation of individual nucleotide positions of the target sites of si88, si117, si123, si128, si135, and si174 across SARS-CoV-2 variants.** The conservation was derived from approximately 200,000 SARS-CoV-2 genome sequences that were retrieved from GISAID and grouped by variant. Conservation is expressed as the percentage of analyzed sequences within each variant that contained the complementary base to the siRNA antisense strand. The conservation is indicated by the color code and % values given in cells.


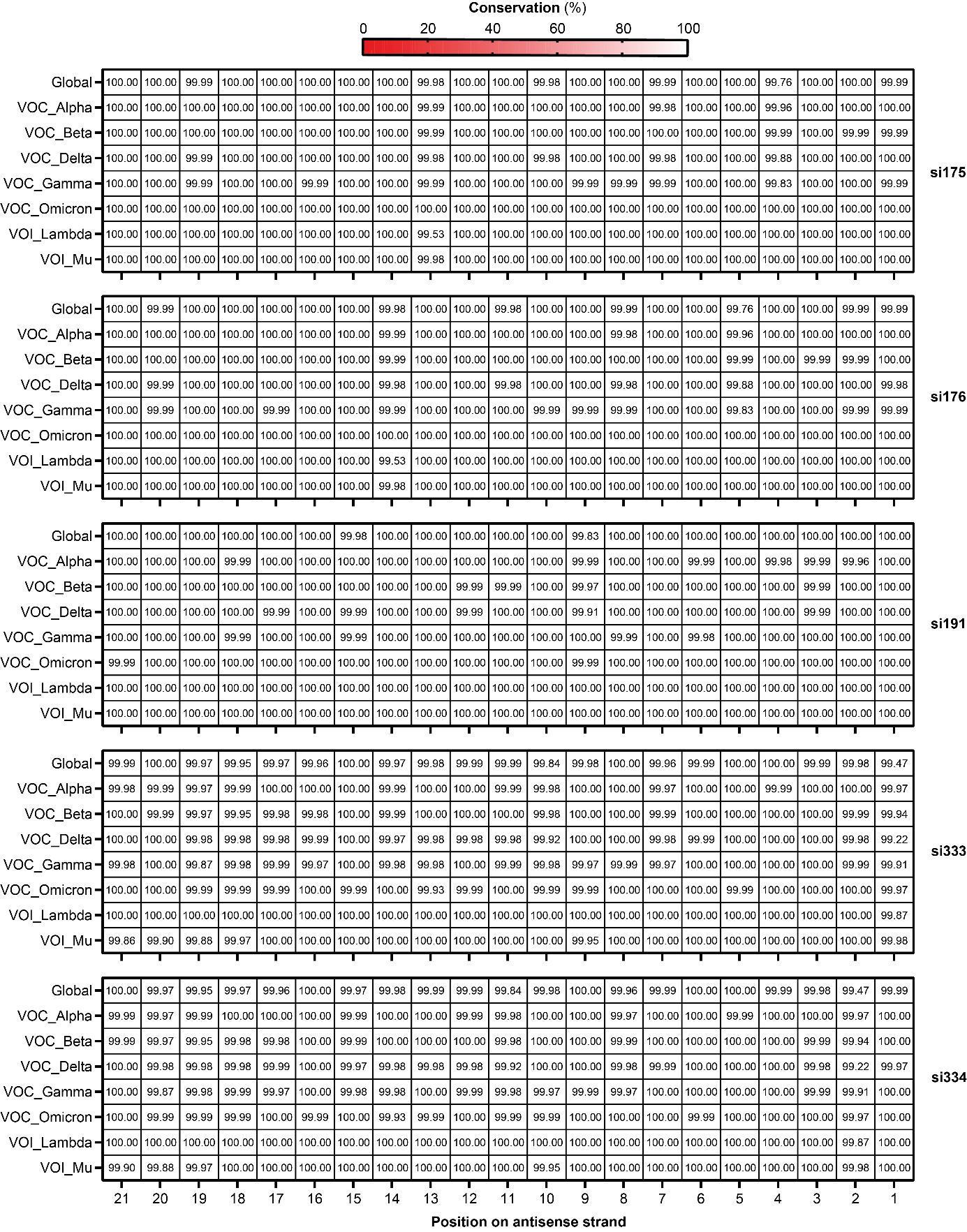


**Fig S7.** **Conservation of individual nucleotide positions of the target regions of si175, si176, si191, si333, and si334 across SARS-CoV-2 variants.** The conservation was derived from approximately 200,000 SARS-CoV-2 genome sequences that were retrieved from GISAID and grouped by variant. Conservation is expressed as the percentage of analyzed sequences within each variant that contained the complementary base to the siRNA antisense strand. The conservation is indicated by the color code and % values given in cells.


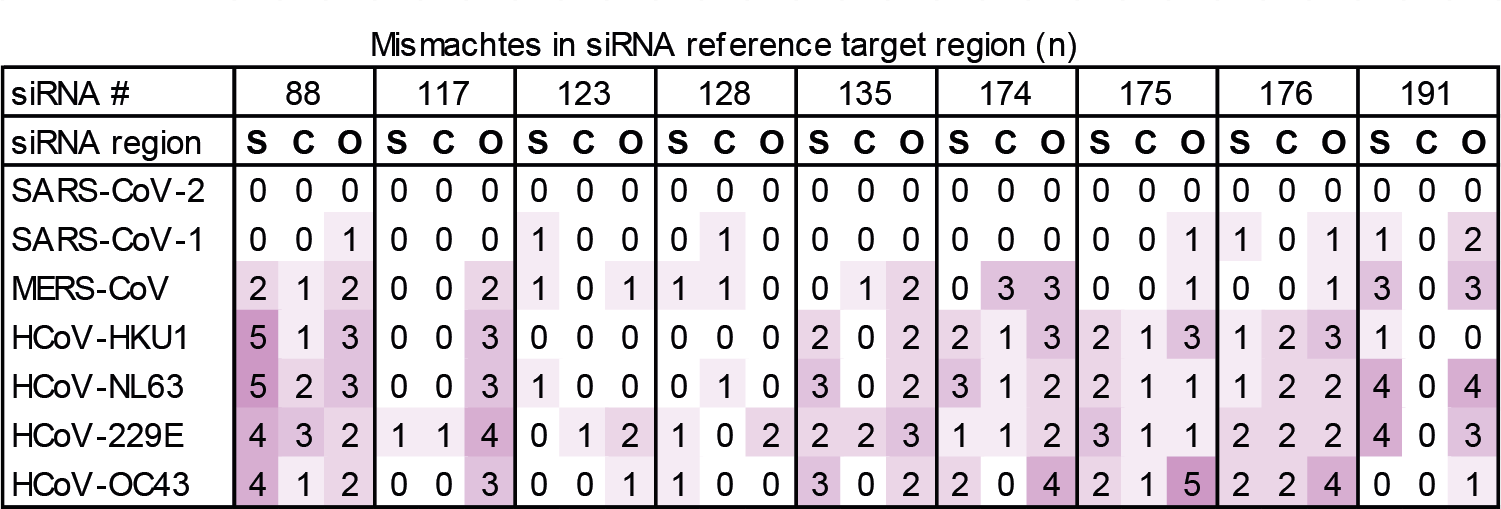


**Fig S8. Mismatch analysis of siRNA target sites in HCoV reference genomes used for the design of luciferase reporters.** The table summarizes the number of mismatches between each siRNA and the corresponding HCoV target sites derived from the reference genome sequences shown in Supplementary Figure S1. Mismatches are grouped according to their position within the siRNA antisense strand as S (seed region, positions 2–8), C (central region, positions 9–12), and O (other positions, position 1 and positions 13–21). Color intensity reflects the total number of mismatches within each category.

**
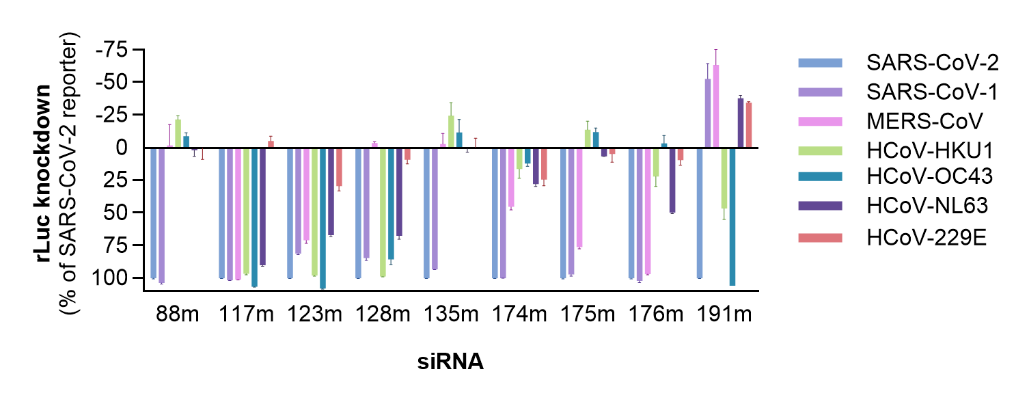
**

**Fig S9. Evaluation of siRNA activity against target site variations derived from HCoVs using dual-luciferase reporters including negative knockdown values.** siRNAs were co-transfected at a dose representing 100 x IC_50_ values, along with plasmids containing the respective version of the siRNA target site in the 3´UTR of *Renilla* luciferase (rLuc). Data were normalized to the knockdown efficiency observed for the SARS-CoV-2 reporter. Data represent mean ± SD (n = 3 to 6).

**
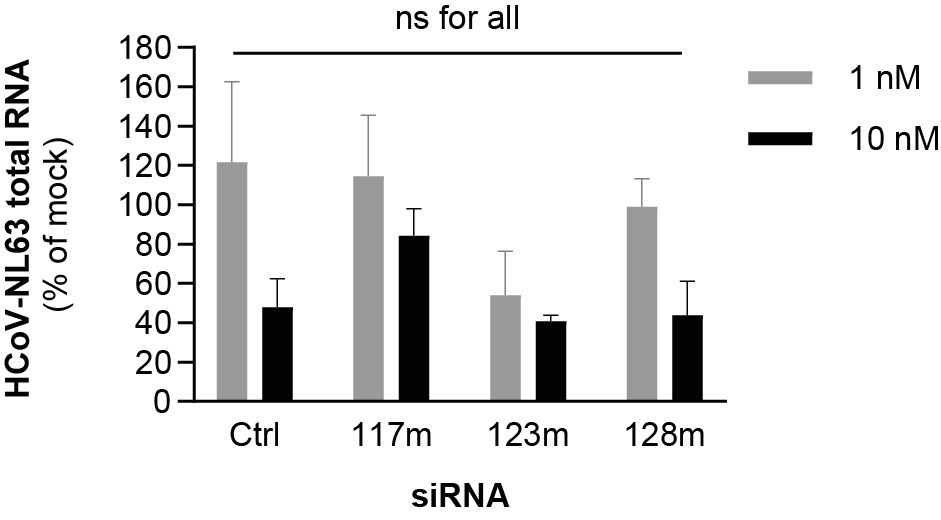
**

**Fig S10. Antiviral activity of selected siRNAs against HCoV-NL63.** Chemically modified siRNA were reverse transfection into Huh7.5 cells at the indicated siRNA concentrations using Lipofectamine RNAiMAX and 4 hours later infected with HCoV-NL63 (MOI = 0.1). Viral replication was quantified by RT-qPCR from cell lysates 48 hours post infection. Bars represent mean ± SD (n=3). Statistical differences between CoV-specific siRNAs and the control siRNA (Ctrl) were calculated using Brown–Forsythe and Welch ANOVA followed by Dunnett’s T3 multiple comparisons test. ns = non-significant

**Table S1. Primer and probe sequences used for RT-qPCR analysis**

| **Primer/Probe Name** | **Target** | **Sequence (5’-3’)** |
| --- | --- | --- |
| OC43-RdRP_fw | HCoV-OC43 RNA-dependent RNA polymerase (Nsp12) | TATGGTGGCTGGGATGATATGTTAC |
| OC43-RdRP_rev | HCoV-OC43 RNA-dependent RNA polymerase (Nsp12) | AGGTTTGGCATAGCACGATCACACTT |
| OC43-RdRP_probe | HCoV-OC43 RNA-dependent RNA polymerase (Nsp12) | FAM- ACAATCCTGTACTTATGGGTTGGGATTATCCT- BHQ1 |
| OC43_NC_fw | HCoV-OC43 nucleocapsid protein | CGATGAGGCTATTCCGACTAGGT |
| OC43_NC_rev | HCoV-OC43 nucleocapsid protein | CCTTCCTGAGCCTTCAATATAGTAACC |
| OC43_NC_probe | HCoV-OC43 nucleocapsid protein | FAM- TCCGCCTGGCACGGTACTCCCT- BHQ1 |
| NL63_NC_fw | HCoV-NL63 nucleocapsid protein | GCGTGTTCCTACCAGAGAGGA |
| NL63_NC_rev | HCoV-NL63 nucleocapsid protein | GCAAGCTGTGGAAAACCTTTGGCA |
| NL63_NC_probe | HCoV-NL63 nucleocapsid protein | FAM- ATGTTATTCAGTGCTTTGGTCCTCGTGAT- TAM |
| SARS1_RdRP_fw | SARS-CoV-1 RDRP | CGTCTGCGGAATGTGGAAAG |
| SARS1/2_RdRP_rev | SARS-CoV-1 RDRP | TAAGACGGGCTGCACTTACA |
| SARS1_N_fw | SARS-CoV-1 nucleocapsid protein | GACCCCAATCAAACCAACGT |
| SARS1_N_rev | SARS-CoV-1 nucleocapsid protein | TCTGGTTATTGTCAGTTGAATCTG |
| SARS2_RdRP_fw | SARS-CoV-2 RDRP | CGTCTGCGGTATGTGGAAAG |
| SARS2_N_fw | SARS-CoV-2 nucleocapsid protein | GACCCCAAAATCAGCGAAAT |
| SARS2_N_rev | SARS-CoV-2 nucleocapsid protein | TCTGGTTACTGCCAGTTGAATCTG |
| 18s_Fw | 18s rRNA | AAACGGCTACCACATCCA |
| 18s_rev | 18s rRNA | CCTCCAATGGATCCTCGT |
| MERS_up-E_fwd | MERS-CoV envelope protein | GCAACGCGCGATTCAGTT |
| MERS_up-E_rev | MERS-CoV envelope protein | GCCTCTACACGGGACCCATA |
| hRPS18_fwd | 18s rRNA | GCGGCGGAAAATAGCCTTTG |
| hRPS18_rev | 18s rRNA | GATCACACGTTCCACCTCATC |

**Table S2. Cycling conditions used for RT-qPCR analysis**

| **Cycle Step** | **Temp.** | **Time** | **Cycle No.** |
| --- | --- | --- | --- |
| **PCR cycling conditions for HCoV-OC43 and HCoV-NL63 RNA** | | | |
| Reverse transcription (RT) | 50°C | 10 min | 1 |
| RT inactivation | 95°C | 3 min | 1 |
| Denaturation | 95°C | 15 s | 45 |
| Annealing/Extension | 60°C | 30 s |  |
| **PCR cycling conditions for SARS-CoV-1 and SARS-CoV-2 RNA** | | | |
| Reverse transcription (RT) | 50°C | 10 min | 1 |
| RT inactivation | 95°C | 3 min | 1 |
| Initial Denaturation | 95°C | 5 min | 1 |
| Denaturation | 95°C | 15 s | 45 |
| Annealing | 55°C | 10 s |  |
| Extension | 72°C | 25 s |  |
| **PCR cycling conditions for MERS-CoV RNA** | | | |
| Initial Denaturation | 95°C | 60 s | 1 |
| Denaturation | 95°C | 15 s | 45 |
| Annealing/Extension | 60°C | 30 s |  |
| Melt Curve | 60-95°C | 15 s | 1 |
